# Osteoporotic osseointegration: therapeutic hallmarks and engineering strategies

**DOI:** 10.7150/thno.96516

**Published:** 2024-06-17

**Authors:** Jiayao Chen, Zhuowen Hao, Hanke Li, Jianping Wang, Tianhong Chen, Ying Wang, Guang Shi, Junwu Wang, Zepu Wang, Zheyuan Zhang, Jingfeng Li

**Affiliations:** 1 Department of Orthopedics, Zhongnan Hospital of Wuhan University, Wuhan 430071, P.R. China.; 2 Department of Obstetrics and Gynecology, Renmin Hospital of Wuhan University, Wuhan 430060, P.R. China.

**Keywords:** Bone tissue engineering, Osteoporosis, Bone regeneration, Engineering stimuli, Applications

## Abstract

Osteoporosis is a systemic skeletal disease caused by an imbalance between bone resorption and formation. Current treatments primarily involve systemic medication and hormone therapy. However, these systemic treatments lack directionality and are often ineffective for locally severe osteoporosis, with the potential for complex adverse reactions. Consequently, treatment strategies using bioactive materials or external interventions have emerged as the most promising approaches. This review proposes twelve microenvironmental treatment targets for osteoporosis-related pathological changes, including local accumulation of inflammatory factors and reactive oxygen species (ROS), imbalance of mitochondrial dynamics, insulin resistance, disruption of bone cell autophagy, imbalance of bone cell apoptosis, changes in neural secretions, aging of bone cells, increased local bone tissue vascular destruction, and decreased regeneration. Additionally, this review examines the current research status of effective or potential biophysical and biochemical stimuli based on these microenvironmental treatment targets and summarizes the advantages and optimal parameters of different bioengineering stimuli to support preclinical and clinical research on osteoporosis treatment and bone regeneration. Finally, the review addresses ongoing challenges and future research prospects.

## 1. Introduction

Osteoporosis is a disease that commonly affects elderly individuals and is characterized by a decrease in bone tissue quality and density, leading to an increased risk of fractures 1. The prevalence of osteoporosis is widespread globally, affecting individuals of all ages. A 2020 epidemiologic analysis conducted in the United States revealed a bimodal distribution of osteoporotic fracture incidence, with peaks occurring in both younger and older adults 2. In the younger age group, the majority of patients are male, possibly due to differences in metabolic rates and lifestyle behaviors. However, in the over-50 age group, female patients outnumber and predominate over male patients. According to statistical data, the United States has approximately 10 million individuals with osteoporosis in the over-50 age group, with an additional 34 million at risk of developing the disease 3. Similarly, China also faces a significant healthcare burden due to osteoporosis. The severe consequences of osteoporosis primarily stem from decreased bone mass, and individuals with lower bone mass are at greater risk of experiencing severe fractures. Fractures of long bones are most common in younger age groups, whereas fractures of the forearms, hips, and vertebrae are more prevalent in older age groups. Moreover, the presence of chronic diseases such as diabetes and renal insufficiency, as well as the long-term use of certain medications such as hormonal drugs, can complicate the treatment of osteoporosis and fractures 4, 5.

In response to the challenging problem of curing osteoporosis, current research on osseointegration and bone regeneration in osteoporosis patients aims to identify new methods and therapeutic means to enhance the proliferation and differentiation ability of bone cells. The objective is to improve the functional restoration and therapeutic efficacy of bone defects. Significant research advancements have been made in areas such as the application of bioactive substances, the development of stem cell technology, and the study of gene therapy.

According to the Guidelines for the Diagnosis and Treatment of Primary Osteoporosis formulated by the Osteoporosis and Bone Mineral Salt Diseases Branch of the Chinese Medical Association, preventive and curative measures for osteoporosis include basic measures, pharmacological interventions, and rehabilitation 6. Basic measures include lifestyle habit changes, calcium and vitamin D supplementation, and other preventive measures to prevent the occurrence of osteoporosis 7. Rehabilitation aims to minimize the occurrence of secondary injuries during the treatment phase and provide a safe environment for rehabilitation. The medication used in the treatment phase is of significant importance, with bone resorption inhibitors, bone formation promoters, bisphosphonates, other mechanism drugs, and proprietary Chinese medicines being the main therapeutic drugs. Among them, bisphosphonates are the most widely used drugs for inhibiting bone resorption. These compounds, which are stabilized analogs of pyrophosphates, have a high affinity for skeletal hydroxyapatite due to the presence of a P-C-P moiety 8. They specifically bind to the active site of bone remodeling, thereby inhibiting osteoclast function and bone resorption. Although bisphosphonates generally have good safety profiles, adverse reactions such as gastrointestinal effects, acute phase reactions, renal impairment, and osteonecrosis of the jaw can still occur. Other drugs that inhibit bone resorption include monoclonal antibodies targeting the receptor activator of nuclear factor-κB ligand (RANKL), calcitonin, and estrogen 9. Bone formation-promoting drugs, as well as bifunctional drugs such as parathyroid hormone analogs, active vitamin D and its analogs, vitamin K, and romosozumab, have been widely recognized for clinical application 10. However, systemic therapy has limitations, such as the need for treatment continuation for more than one year and the risk of osteoporotic fractures before and after discontinuation of treatment. For instance, oral bisphosphonate treatment is generally recommended to last for more than 5 years; the decision to continue treatment is based on efficacy, meaning that patients may face up to 10 years of treatment. Currently, calcium or vitamin D supplementation is recommended as the basic medication along with antiosteoporosis treatment. However, the excessively long duration of treatment and the difficulty in claiming a significant therapeutic effect often result in low patient compliance and high treatment costs. Therefore, it is essential to develop localized osteoporosis treatment strategies that are independent of systemic therapy. The objective is to design combinations that effectively repair localized damage and provide rapid onset of action.

The pathogenesis of osteoporosis involves the disruption of bone homeostasis, which refers to the balance between bone resorption by osteoclasts and bone production by osteoblasts (OBs). Numerous studies have shown that osteoblast differentiation and function are controlled by various transcription factors (e.g., steroids and runt-related protein 2 (Runx2)) and major developmental signals (e.g., Wingless and INT-1 signaling) 11. Similarly, the regulation of osteoclast differentiation and/or function is influenced by factors such as RANKL, macrophage colony-stimulating factor (M-CSF), cytokines, and αVβ3 integrins 12. Moreover, extensive research has been conducted on genes associated with the pathogenesis of osteoporosis, with hundreds of gene loci related to osteoporosis being identified. Examples of these genes include low-density lipoprotein receptor-related protein 5 (LRP5), estrogen receptor α (ESR1), and osteoprotegerin (OPG), which all play a role in the regulation of bone homeostasis 13, 14. This knowledge can contribute to the development of personalized treatments for osteoporosis. There is great potential for developing new therapeutic strategies that target osteoclastogenesis, osteoblastogenesis, or other aspects of bone homeostasis maintenance 15. In particular, the use of materials with high osteogenic and osteoconductive activity has emerged as a promising alternative for treating osteoporosis. Over the past few years, injectable hydrogels and nanomaterials have gained recognition as promising biomaterials for osteoporosis treatment 16. To enhance the accuracy and sustainability of drug release, researchers have extensively studied local delivery systems based on injectable hydrogels and drug-loaded nanoparticles. Furthermore, nanomaterials with inherent therapeutic activity, such as ROS clearance, acid neutralization, or immunomodulatory hydrogels, have shown the ability to effectively regulate bone homeostasis and reverse osteoporosis 17. It is important to note that the categories of materials and technologies mentioned above do not directly affect bone tissue. Rather, it is the engineered stimuli they generate, including both biophysical and biochemical stimuli, that have an impact. This review also classifies biophysical stimuli into two categories: endogenous and exogenous stimuli. This categorization helps differentiate the sources and applications of various technologies.

**Scheme 1:**
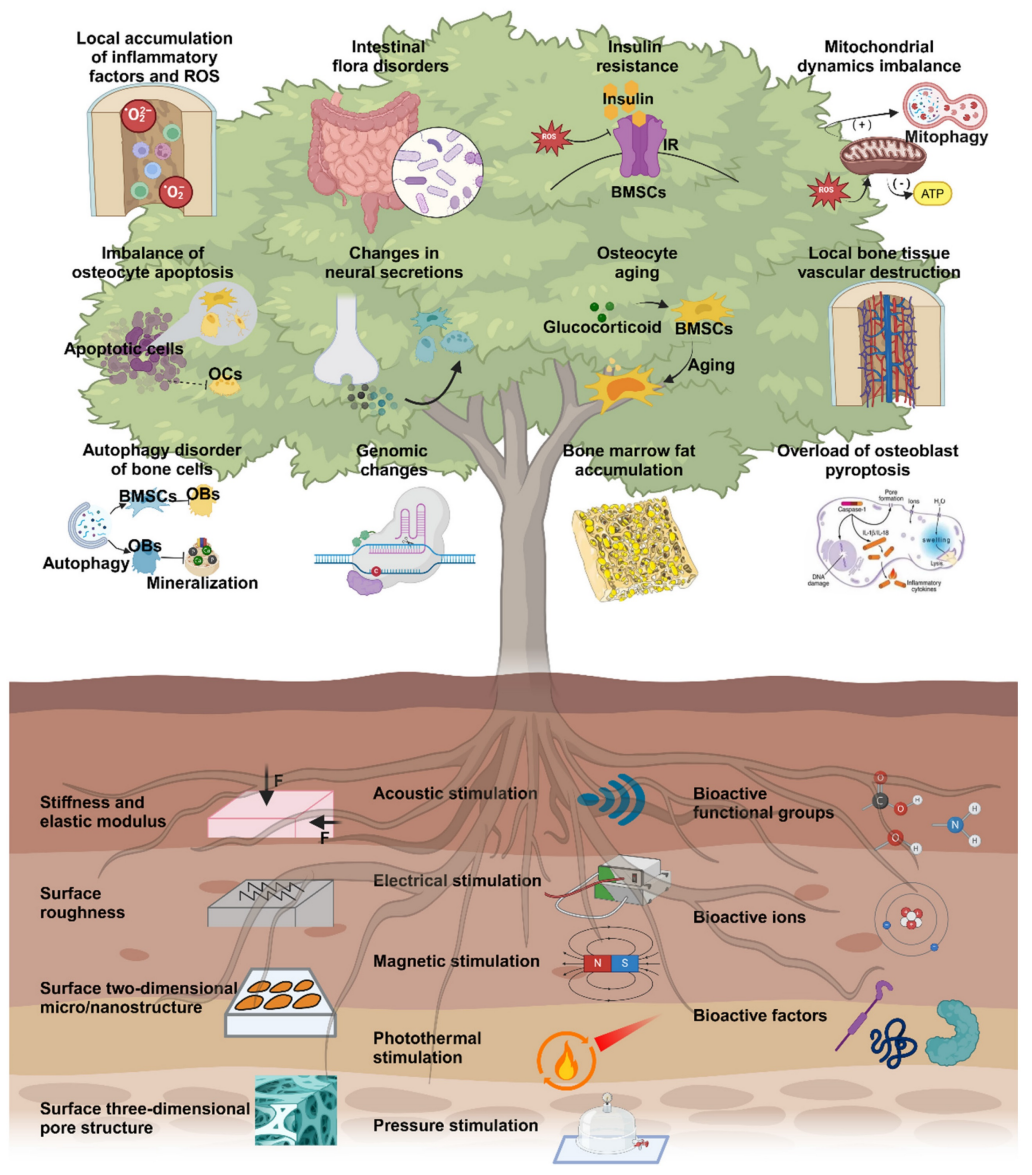
Overview of microenvironmental hallmarks of osteoporosis and engineering stimulations in topical osteoporotic osteoregeneration and osseointegration. Created with BioRender.com.

In this review, we present twelve microenvironmental hallmarks for topical therapy and provide an overview of common biophysical and biochemical stimuli used in clinical practice and related research. The focus is on investigating the impact and potential application of various engineering stimuli on these twelve microenvironmental hallmarks (**Scheme 1**). Based on this, we summarize the optimal parameters that have been proven or have the potential for application in individual engineered stimuli. By considering these specific targets and the functions of engineering stimuli, the development of targeted treatment strategies for osteoporosis can benefit the management of localized severe diseases and enable individualized patient treatment, with significant future potential.

## 2. Microenvironmental hallmarks of osteoporosis

The pathogenesis of osteoporosis includes various aspects, including cellular behavior, the tissue microenvironment, and hormone secretion 18. Here, we propose twelve microenvironmental hallmarks specific to osteoporosis (**Scheme** 1). These hallmarks differ from those identified in previous studies on common mechanisms and focused on the pathological processes that directly influence the development of osteoporosis in terms of pathogenesis and pathological outcomes. There are two reasons for these specific markers of osteoporosis. First, these changes can be observed or quantified through experiments or other detection methods. Second, targeted treatments and intervention measures have been demonstrated to delay and improve the pathological process of osteoporosis or show the potential to do so. Osteoporosis is a complex systemic process, and investigating its underlying mechanisms and pathogenesis hallmarks can contribute to the development of focused treatment strategies in a singular direction 19, 20. Consequently, utilizing different hallmark agents as novel entry points for future antiosteoporosis treatments is necessary to achieve personalized and effective management of osteoporosis.

### 2.1 Local accumulation of inflammatory factors and ROS

**Figure 1:**
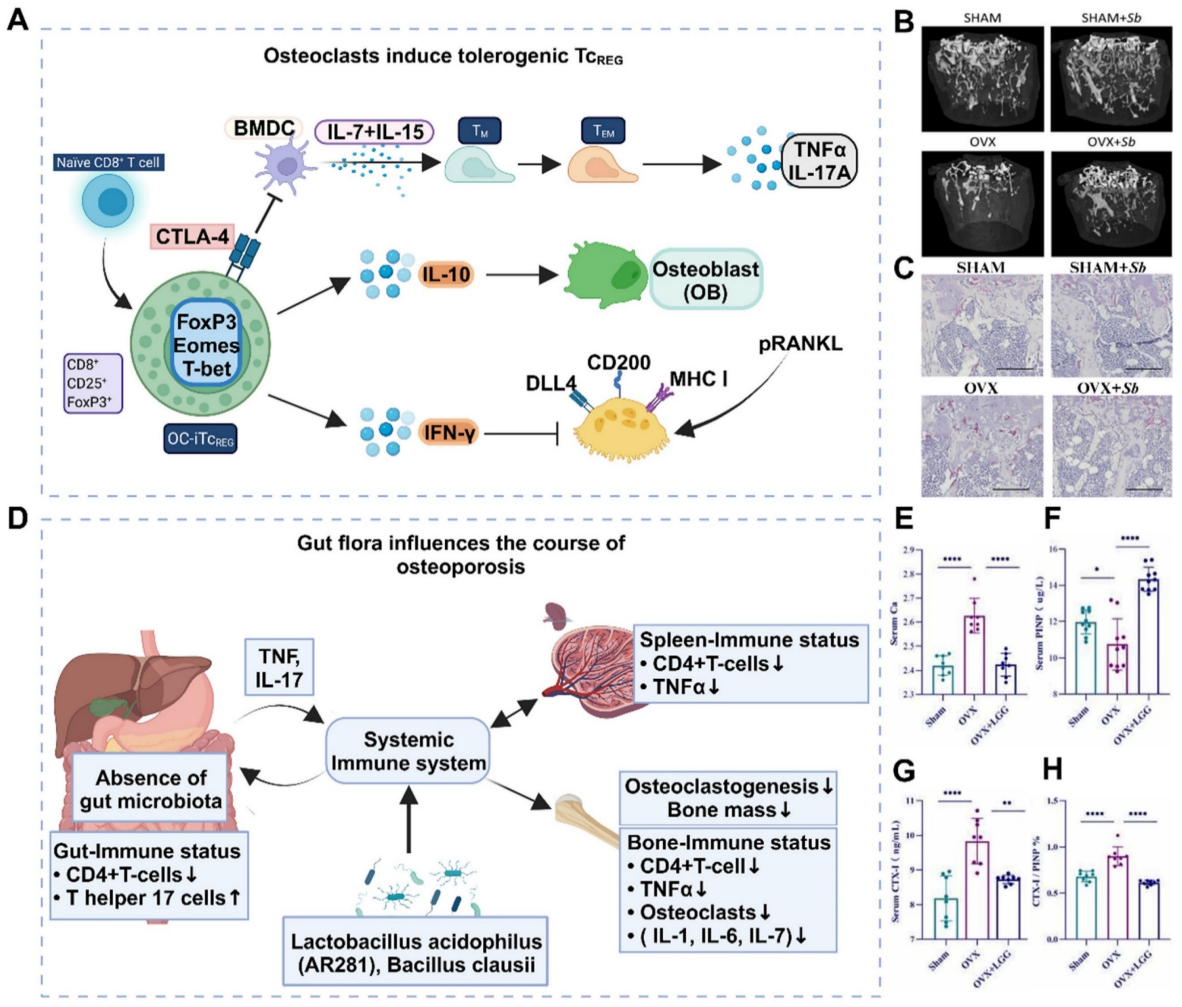
Local accumulation of inflammatory factors and ROS. (A) Mechanisms of growth and differentiation of inflammatory cells induced by the osteoporotic microenvironment. (B-C) (B) Representative microcomputed tomography images of femurs from SHAM mice and oophorectomy (OVX) mice (± antimony administered). (C) Histological analysis of OCLs from tibias from SHAM and OVX mice treated or not treated with Sb determined via TRAcP staining (in purple). Scale bars: 100 µm. Adapted with permission from 36, copyright 2023. (D) The gut flora influences the osteoporosis process through the immune system. Created with BioRender.com. (E-H) Probiotic LGG administration improved the expression of bone turnover markers and changes in serum Ca levels, CTX-I, PINP and CTX-I/PINP in all groups. (n = 8-10). Adapted with permission from 32, copyright 2023.

The inflammatory response is a well-established pathological change observed in osteoporosis, particularly among postmenopausal women. This is commonly attributed to estrogen deficiency and alterations in the gut microbiota. However, further research is necessary to understand the interplay between these factors and their sequential occurrence. The inflammatory response serves as an innate defense mechanism against external stimuli and cellular damage and involves the infiltration of inflammatory cells and the release of inflammatory factors 21. Excessive inflammation is detrimental to the human body and is considered a primary contributor to the development and progression of osteoporosis. In situations of cell necrosis or excessive apoptosis, there is an overproduction of inflammatory factors (**Figure** 1A) 22. Estrogen deficiency is a prevalent cause of osteoporosis and has been implicated in the regulation of cell apoptosis 23. The estrogen receptor (ER) response in OBs activates Wnt/β-Signal transduction, resulting in a reduction in the secretion of bone resorption-promoting molecules such as interleukin-6 (IL-6) and FasL, as well as RANKL, whereas increasing the secretion of OPG 24. The ER response to estrogen in osteoclasts regulates RANK information transduction levels and enhances osteoclast apoptosis levels. Consequently, patients with estrogen deficiency, particularly postmenopausal females, experience reduced inhibition of osteoclast function, leading to intensified bone resorption, increased bone cell apoptosis, and heightened production of inflammatory factors such as tumor necrosis factor-α (TNF-α) 25. The accumulation of local inflammatory factors such as IL-1, IL-6, RANKL, and IL-17 may facilitate osteoclast activation, exacerbate osteoporosis progression and contribute to severe localized pain in patients 26.

Effective clearance of local inflammatory factors is advantageous for mitigating disease progression, alleviating patient pain, and enhancing patient treatment compliance. (**Figure** 1B-C). Like the accumulation of inflammatory factors, local ROS accumulation can also impede implant stability and the bone repair process 27. ROS promote the activation of osteoclasts and can be produced by osteoclast precursor cells in the body. This signaling cascade involves nicotinamide adenine dinucleotide phosphate oxidase 1 (Nox1) and Ras-related C3 botulinum toxin substrate 1 (Rac1) 28. A deficiency of Gp91phox, the plasma membrane subunit of Nox1, can lead to defects in osteoclast differentiation, which can be reversed by H_2_O_2_, suggesting that ROS may play a crucial role in osteoclast generation 29. The downstream target of ROS in RANKL-mediated signal transduction has not been determined, but an increase in oxidative stress may be triggered by the activation of B cells. Nuclear factor κ light chain enhancers and mitogen-activated protein kinase (MAPK) signaling pathways enhance the cellular activity of osteoclasts 30. Conversely, the protective effects of antioxidant enzymes such as NADPH oxidase, heme oxygenase-1 (HO-1), superoxide dismutase, and various mitochondrial oxidases have been demonstrated, indicating that they may inhibit osteoclast formation by neutralizing ROS 31.

### 2.2 Intestinal flora disorders

Osteoporosis is a systemic disease characterized by chronic low-grade inflammation. Immune cells, cytokines, and chemokines play crucial roles in osteoporosis, with the immune system's effect on bone remodeling often manifested through B and T-cell activation, increased IL-17, IL-6, RANKL, TNF-α, and other pro-bone resorption factors (**Figure** 1D). The expression of IL-17, IL-6, RANKL, TNF-α, and other pro-bone resorption factors was found to increase 32. The intestinal flora primarily regulates bone metabolism by inducing changes in various aspects of the immune system.

Estrogen deficiency increases intestinal permeability, resulting in the activation of T cells by intestinal microbial components and leading to an increase in TNF+ T cells and Th17 cells in the intestinal wall. This, in turn, increases the production of TNF-α and IL-17 in the lamina propria of the intestinal wall 33. Lactobacillus acidophilus and Bacillus clausii have been shown to inhibit deovulation-induced bone loss in mice by modulating the balance between Treg-Th17 cells. Bacillus clausii acts by modulating the balance between Treg-Th17 cells, decreasing the levels of proinflammatory cytokines (IL-6, IL-17, and TNF-α) and increasing the levels of anti-inflammatory cytokines (IL-10 and IFN-γ) 34. The gut flora also plays a significant role in the pathogenesis of postmenopausal osteoporosis. The levels of small intestinal proinflammatory cytokines (including IL-1, IL-6, TNF-α, and IL-17) were significantly greater in nulliparous mice than in normal mice, whereas oral gavage of Lactobacillus shortcombii AR281 in nulliparous mice significantly suppressed the levels of IL-1, IL-6, TNF-α, and IL-17 (**Figure** 1E-H) 35. Proinflammatory osteoclasts primarily affect bone resorption through the RANKL/OPG pathway. Bifidobacterium reduced the concentration of lipopolysaccharide and resulted in the downregulation of the TLR-4/p-NF-κB/TNF-α inflammatory pathway, thereby improving lumbar bone loss in ovariectomized mice 35.

### 2.3 Mitochondrial dynamics imbalance

Upregulation of ROS levels in the bone microenvironment is one of the main pathological changes observed in osteoporosis. High ROS levels can disrupt the mitochondrial membrane potential, alter mitochondrial structure, and impact the tricarboxylic acid (TCA) cycle 37. Damaged mitochondria tend to undergo mitochondrial autophagy, and recent research has shown that this process promotes the development of several diseases. In a study of osteoporosis associated with type 2 diabetes, the activation of mitochondrial autophagy was found to inhibit the activity of OBs through various processes produced during mitochondrial autophagy *in vitro* or in animal models, resulting in bone loss 38. Additionally, Yang *et al.* reported that hypoxia can induce mitochondrial dysfunction in OBs and stimulate mitochondrial autophagy, leading to bone loss in a rat model of apical periodontitis 39.

**Figure 2:**
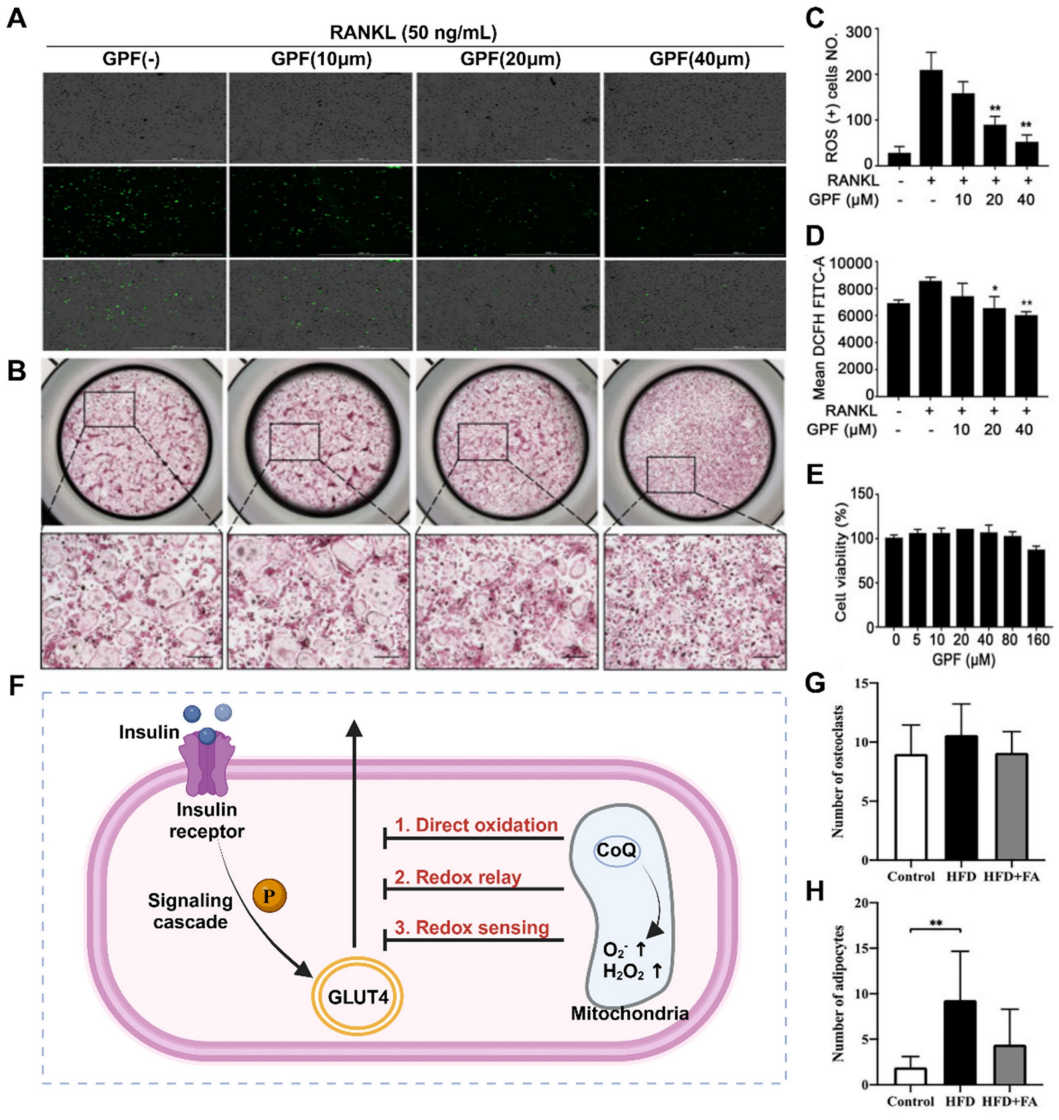
Mechanisms and experiments related to ROS clearance (A, B, C) GPF attenuates RANKL-induced ROS generation *in vitro*. (D) Representative images showing the inhibitory effect of GPFs on BMM osteoclastogenesis. BMMs were incubated with RANKL and M-CSF in the absence or presence of GPFs (0, 10, 20 and 40 μM). On day 7, the cells were fixed, and the cells were stained for TRAP (n = 3). (E) CCK-8 assay after 96 h of treatment with different concentrations of GPF (n = 3). Adapted with permission from 45, copyright 2021. (F) Mechanisms associated with the development of osteoporosis and insulin resistance. Created with BioRender.com. (G-H) Folic acid ameliorated bone loss and destruction induced by a high-fat diet in mice, and quantitative statistics of HE-stained images showed a reduction in the number of osteoclasts and adipocytes in the HFD+FA group compared with the HFD group. Adapted with permission from 33, copyright 2021.

Treatment of mitochondrial dysfunction has been shown to be beneficial for bone regeneration, whereas restoring mitochondrial function can help slow the progression of osteoporosis. Zhang *et al.* prepared a poly (succinic acid glycerol) scaffold that promotes bone regeneration by producing metabolic intermediates and normalizing the mitochondrial membrane potential of marrow mesenchymal stem cells (MSCs) 40. Hang *et al.* created a 3D-printed and bioenergy-active double network hydrogel containing sodium polyphosphate, which enhances bone regeneration by promoting cell metabolism and providing high-energy phosphate bonds such as ATP 41. The association between mitochondrial dynamics and osteoporosis has led to new intervention strategies with promising potential for future osteoporosis treatment (**Figure** 2A-E).

### 2.4 Insulin resistance

In the past, it was commonly believed that there was a direct causal association between diabetes and osteoporosis, with diabetic patients being more susceptible to bone loss. However, recent research has revealed that osteoporosis exacerbates insulin resistance (**Figure** 2F), possibly due to elevated levels of ROS in the osteoporosis microenvironment. Elevated ROS levels can inhibit the downstream signaling cascade of insulin receptors and phosphatidylinositol-3 kinase (PI3K), leading to insulin resistance and glucose metabolism disorders in Bone marrow mesenchymal stem cells (BMSCs) 42. Insulin resistance in bone tissue hampers glucose uptake and increases blood glucose levels, thereby impairing Runx2 activity and osteogenic differentiation. In addition, reduced glucose uptake diminishes acetyl-CoA levels and the TCA cycle, which play crucial roles in the posttranslational acetylation and transcriptional activity of osterix and Runx2 43. Importantly, disrupted glucose metabolism caused by insulin resistance reduces the energy available for physiological processes such as osteoblast differentiation, bone-related protein secretion, and biomineralization. This inhibition of the osteogenic process indirectly leads to increased osteoclast levels, bone loss, and ultimately, osteoporosis 44. Thus, clearance therapy targeting ROS in insulin resistance can attenuate the progression of osteoporosis by improving ATP production and mineralization ability but can also positively impact mitochondrial function recovery.

### 2.5 Autophagic dysfunction in bone cells

**Figure 3:**
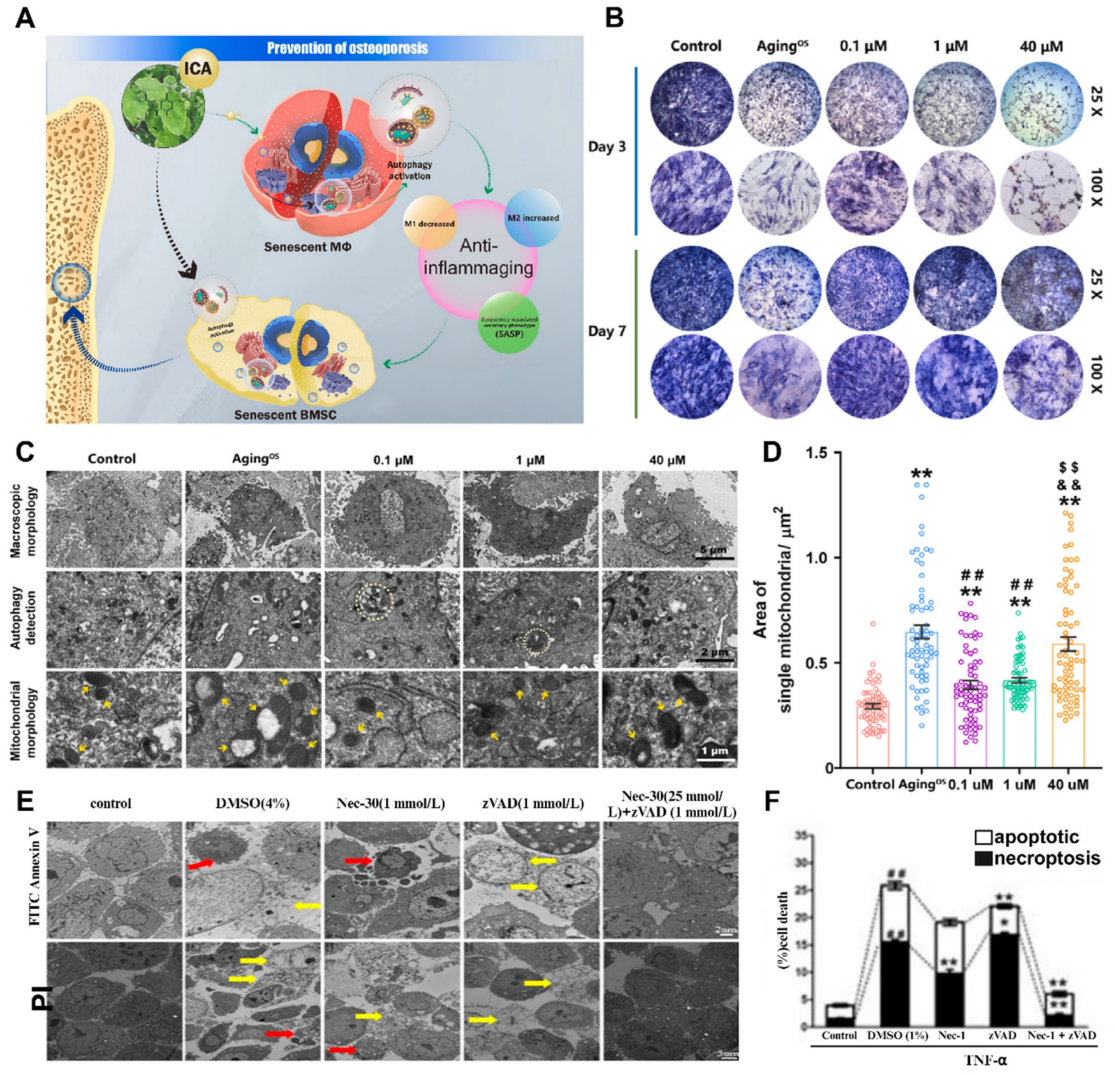
Interaction of cellular autophagy and apoptosis with the osteoporotic microenvironment (A) Schematic illustration of autophagy ultimately relieving osteoporotic bone destruction in diseased cells. (A-D) (B) ICA promoted the osteogenic viability of BMSCs by restoring autophagy according to the results of the osteogenic viability assay and the qualitative and quantitative results of ALP staining after 3 and 7 days of incubation with ICA, respectively (n = 3). (C) TEM observation of autophagosomes (white dashed line) and mitochondria (yellow arrows) in BMSCs after incubation with ICA for 3 days. (D) Quantification of the mitochondrial number and individual mitochondrial area in the different groups. Adapted with permission from 55, copyright 2023. (E, F) TNF-α-induced necroptosis and apoptosis of MLO-Y4 cells. TEM images of osteocytes pretreated for 30 min with DMSO (1%), Nec-1 (30 mmol/L), zVAD (25 mmol/L), or Nec-1 (30 mmol/L) + zVAD (25 mmol/L) and then treated with TNF-α (100 ng/ml) for 24 h. Adapted with permission from 56, copyright 2021.

Autophagy is an essential stress-induced cellular catabolic process that occurs in the physiological environment and plays a crucial role in maintaining cellular and tissue microenvironment homeostasis throughout the human body's entire life cycle. Disruptions in the balance of autophagy have various effects on bone cells, such as BMSCs and OBs, and impact the progression of osteoporosis. The combined effect of autophagy in multiple types of bone cells ensures the vitality of these cells and facilitates damage repair through continuous bone remodeling. Consequently, changes in autophagy levels are closely associated with the onset of osteoporosis (**Figure 3**A-D).

The disruption of autophagy in cells is a direct contributor to osteoporosis 46. BMSCs are pluripotent stem cells that can differentiate, and autophagy disorders that cause abnormal differentiation of BMSCs play a significant role in the pathogenesis of osteoporosis. The excessive production of ROS leads to cellular oxidative stress, which results in cellular aging and damage and impacts the vitality of osteogenic differentiation 47. Insufficient autophagy prevents the timely clearance and recycling of aging or abnormal cells, thereby reducing the energy available for early osteogenic differentiation in BMSCs. However, normal autophagy safeguards undamaged or newly formed bone cells by degrading damaged organelles or aging cells, protecting them from cytotoxic effects 48. Studies have demonstrated that autophagy disorders in OBs negatively impact bone mineralization. *In vitro* experiments in which the key genes ATG5, ATG7, and Beclin-1 were deleted during the autophagy process in OBs have proven to decrease bone mineralization levels and reduce bone density 49. Consequently, adjusting bone cell autophagy and restoring the functions of BMSCs and OBs are potential treatment strategies for effectively reducing bone loss in osteoporosis patients.

### 2.6 Imbalance of osteocyte apoptosis

Apoptosis is a regulated cellular process used by the body to maintain internal equilibrium and promote homeostasis. However, an imbalance in bone cell apoptosis, influenced by specific factors, can significantly disrupt the bone microenvironment and metabolism, leading to the development of osteoporosis. Excessive production of ROS and inflammatory mediators in osteoporosis can cause osteoblast apoptosis to increase, whereas estrogen deficiency weakens the ability to promote osteoclast apoptosis 50. In OBs, exogenous apoptosis signaling is mediated by tumor necrosis factor receptor (TNFR) and death receptor (Fas), both of which are present on the activated osteoblast membrane 51. Estrogen treatment reduces TNFR-induced osteoblast apoptosis via heat shock protein 27 (HSP27) but promotes osteoclast apoptosis through the Fas pathway, thus inhibiting bone resorption and improving the progression of osteoporosis (**Figure** 3E-F).

In addition to exogenous apoptosis, osteoporosis involves various endogenous signaling pathways associated with mitochondrial pathway apoptosis, such as the PI3K/Akt, ERK5, JNK, Wnt/β-catenin, and nuclear factor kappa-B (NF-κB) pathways 52. These pathways, along with P38 and other signaling pathways, interact and promote the expression of crucial targets such as caspases and Bcl-2 family proteins in the mitochondrial pathway, leading to increased osteoblast apoptosis. Treatments targeting mitochondrial pathways can be classified into two types: the first aims to maintain a balance between OBs and osteoclasts by increasing osteoclast apoptosis, whereas the second focuses on inhibiting osteoblast apoptosis 53. OPG generated by OBs plays a role in inducing osteoclast apoptosis by triggering the release of cytochrome from mitochondria in osteoclasts and their precursor cells. This process activates caspase-3 and caspase-9 and facilitates the nuclear translocation of apoptosis-inducing factor (AIF) and mitochondrial endonuclease G (ENDOG), resulting in osteoclast apoptosis and inhibition of osteoclast differentiation and activity 54. Bone marrow mitochondrial protein (OPA) is a transmembrane protein found in mitochondria, and the downregulation of OPA has been shown to inhibit osteoblast apoptosis and ameliorate osteoporosis by increasing ATP production and inhibiting the P38 signaling pathway.

### 2.7 Increased osteoblast pyroptosis

**Figure 4:**
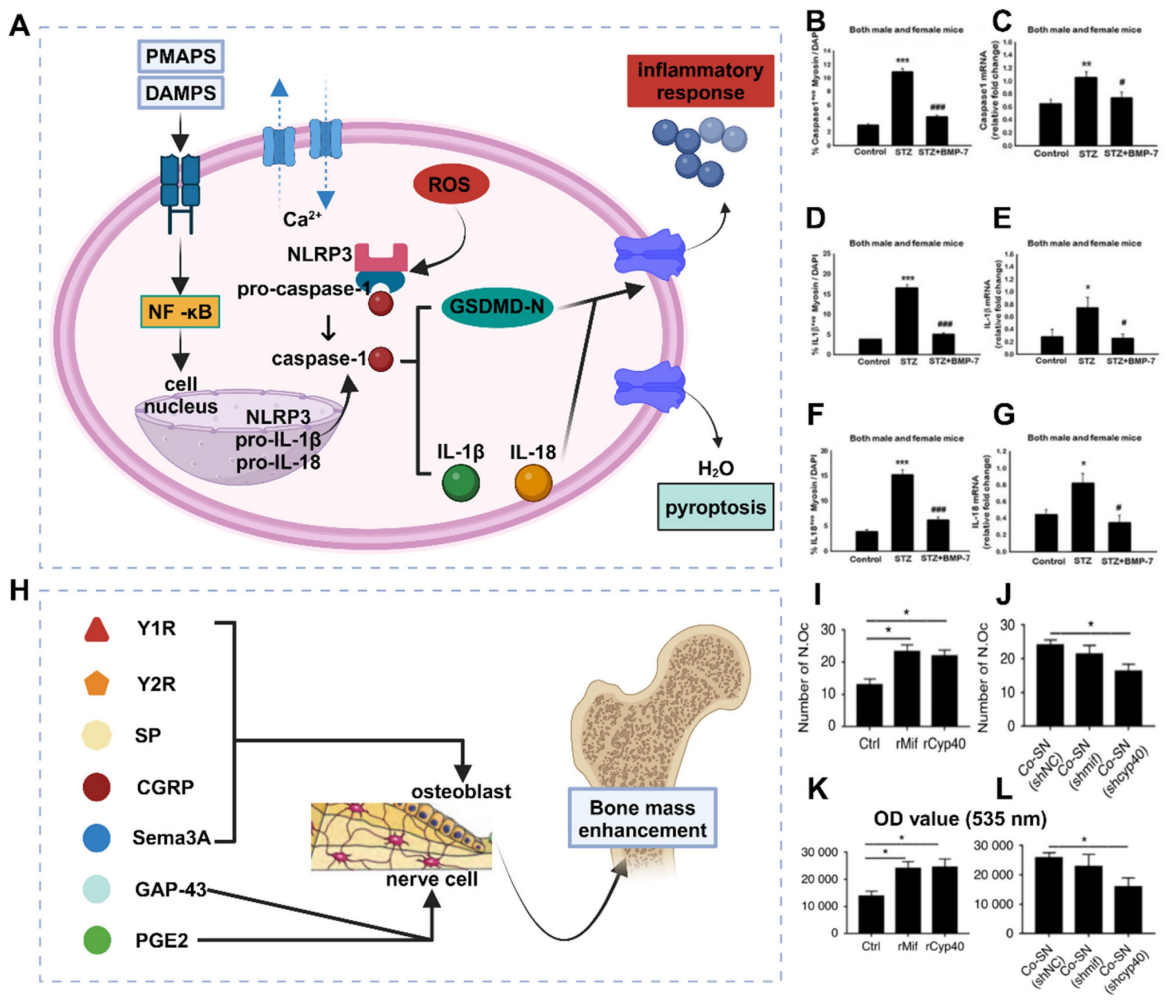
(A) Osteoblast pyroptosis affects the development of osteoporosis by interfering with the release of immune factors. (B-G) Bone morphogenetic protein-7 (BMP-2) administration reduces the cellular cascade markers of cellular pyroptosis caspase1, IL-1β, and IL-18. (B, C) caspase-1, (D, E) IL-1β, (F, G) IL-18. Adapted with permission from 70, copyright 2021. (H) Neurosecretory agents act on neurons and OBs during osteoporosis development. Created with BioRender.com. (I-L) Cyp40 is critical in promoting neurogenesis in bone tissue repair. (I, J) Scoring of TRAP staining of OBs and TRAP-positive multinucleated cells with ≥3 nuclei per well (n=3). (K, L) Resorptive activity was measured by planking BMMC on fluorescent calcium phosphate-coated plates. Adapted with permission from 71, copyright 2023.

Cellular pyroptosis is a novel mechanism of programmed cell death that involves different morphological features and molecular mechanisms than cell autophagy, apoptosis, and necrosis. The activation of cellular pyroptosis leads to the release of inflammatory factors and inflammatory vesicles, consequently triggering local or systemic inflammatory responses (**Figure** 4A). Controlled cellular pyroptosis is beneficial for effectively eliminating pathogens from the body. However, excessive cellular pyroptosis can result in an intense inflammatory response and worsen disease pathology 57. Recent studies have revealed the involvement of osteoblast pyroptosis in the immune defense response of bone tissue, with excessive cellular pyroptosis leading to more severe bone damage. The association between cellular pyroptosis and the development of osteoporosis suggests its potential role in mediating this condition.

The primary function of cellular pyroptosis is to induce a robust inflammatory response and aid the host in combating pathogen infections. The pyroptosis pathway is a significant target for therapeutic interventions and plays a critical role in various diseases, including but not limited to tumors, sepsis, heart disease, diabetes, liver disease, Alzheimer's disease, human immunodeficiency virus (HIV), and gout. Although the direct association between cellular pyroptosis and osteoporosis is not entirely clear, all osteoblastic phenomena are closely associated with inflammation. Inflammatory factors such as IL-1β and IL-18 contribute to osteoporosis pathogenesis through caspase-activated cellular pyroptosis 58. Under inflammatory conditions, NLRP3 inhibits OBs through cellular pyroptosis, and the expression levels of pyroptosis-related proteins are influenced by various traditional Chinese medicines 59. Therefore, the close association between cellular pyroptosis and osteoporosis suggests that cellular pyroptosis leads to increased osteoclast damage, resulting in progressive osteoporosis and a heightened fracture risk. Additionally, NLRP3, GSDMD, IL-1β, IL-18, and caspases 1/4/5/11 are being investigated as potential drug targets due to their essential roles in the pyroptosis pathway 60. Osteoporosis occurs when osteoclast formation surpasses that of OBs, resulting in an imbalance between excessive bone resorption and remodeling. Inflammatory vesicle activation-induced pyroptosis in osteoclasts and OBs might play a crucial role in the pathogenesis of osteoporosis.

### 2.8 Changes in neural secretions

Neurotransmitters and neuropeptides play crucial roles in neural secretions, and changes in their composition and quantity can have a significant impact on the pathological process of osteoporosis (**Figure** 4B). Dysfunction of neurotransmitters can increase psychological stress and stress in patients by affecting signal transmission in the nervous system, thereby influencing bone regeneration and bone resorption processes 61. Although mental illness and osteoporosis are two different systemic diseases, they share significant overlapping factors, such as glucocorticoids, catecholamines, and insulin-like growth factors. Therefore, disorders of neurotransmitters can lead to immune and endocrine system dysfunction and expedite the process of osteoporosis and the occurrence of trauma 62. Several studies have established a chronic mild stress model in rodents to investigate the association between neurotransmitters and osteoporosis 63. These studies revealed symptoms of depression, a decrease in the number of OBs, aggravated bone loss, and reduced bone regeneration. Another pathway associated with neurotransmitter disorders involves catecholamines, which can induce the growth of OBs and osteoclasts 64. Activation of adrenergic receptors increases the expression of RANKL and leads to increased differentiation of osteoclasts, disrupting the balance of bone resorption.

Numerous neurological diseases have been found to be associated with osteoporosis. Patients with Alzheimer's disease have been shown to have greater rates of bone loss and fractures 65. Studies have revealed that an abnormally activated central serotonin regulatory pathway occurs in this disease, resulting in high levels of sympathetic nerve signaling, which activates bone cell β-AR receptors and enhances bone resorption 66. Cholinergic signals may also be implicated in osteoporosis. Current research has indicated significant reductions in the levels of mAChR M5 and mAChR M3 in osteoporosis rat models, providing reliable evidence for the involvement of the AchR signaling pathway in the development of osteoporosis.

Neuropeptides, such as substance P, calcitonin gene-related peptides, and neuropeptide Y (NPY), have been identified as potential regulators of bone metabolism 67. These substances are typically synthesized in the sympathetic nervous system and released into nerve endings surrounding bone and periosteal tissues. They play a crucial role in the pathogenesis of osteoporosis and associated chronic pain. The NPY signaling pathway is mediated by five receptors (Y1R, Y2R, Y4R, Y5R, and Y6R). Y1R and Y2R are involved in the regulation of bone mass 68. However, in the central nervous system, hypothalamic NPY may inhibit bone formation through Y2Rs. The specific mechanism by which NPY affects the behavior of BMSCs during the onset of osteoporosis remains unknown. In a study, it was reported that bone cells secrete excess NPY, which promotes adipogenic differentiation and inhibits osteogenic differentiation of BMSCs. The absence of NPY in bone cells can lead to a high bone mass phenotype and delay aging, reducing bone loss induced by OVX in mice. In a control group of mice, the clinical use of the ANS modulator oryzanol significantly increased bone formation and reversed aging. However, this effect was not observed in mice lacking the bone cell NPY 69. In summary, elevated levels of neuropeptide Y can disrupt normal bone metabolism and osteogenesis by promoting the differentiation of BMSCs into adipocytes, ultimately leading to the development of osteoporosis.

### 2.9 Osteocyte aging

**Figure 5:**
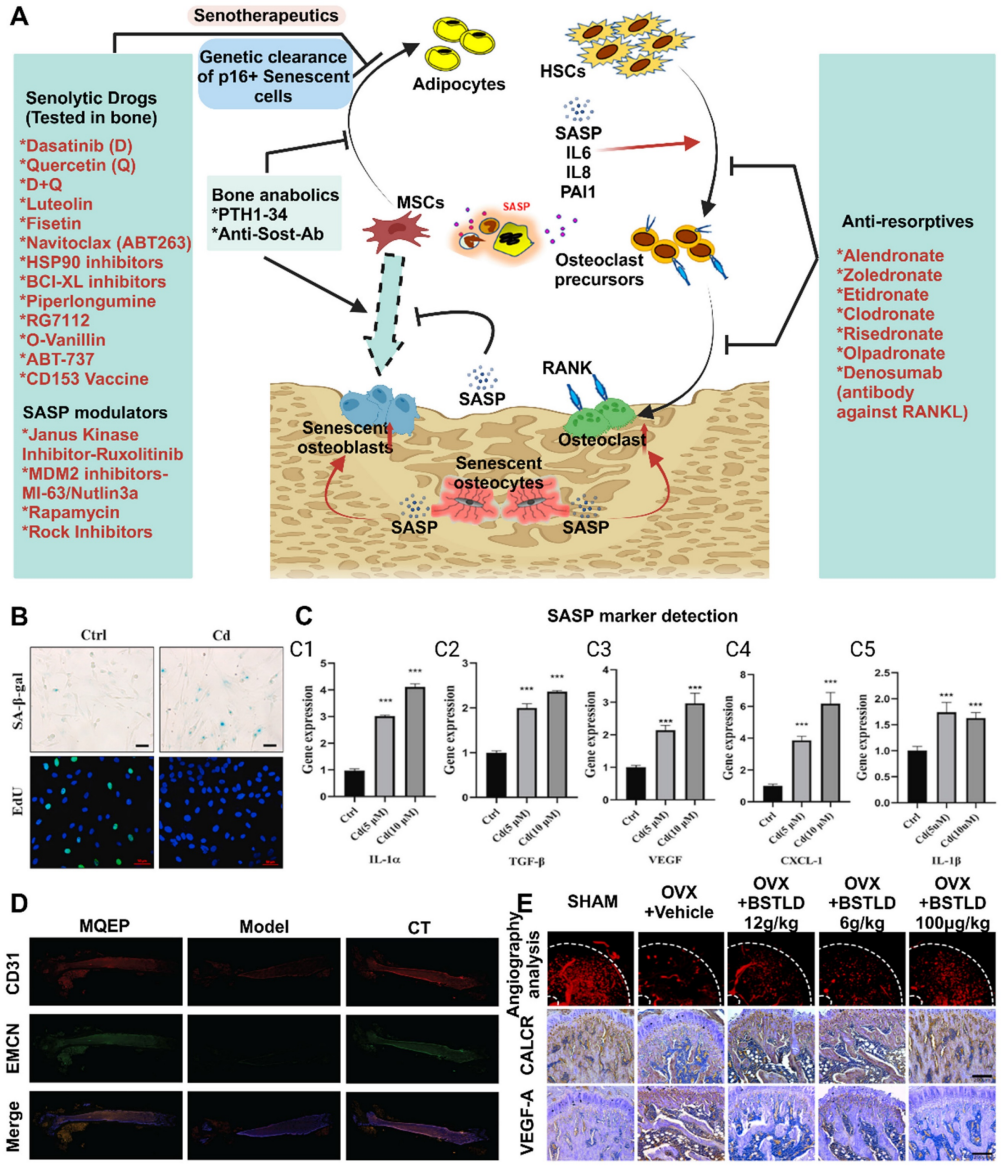
Advances in the study of cellular senescence and local tissue vascularization. (A) Mechanisms of skeletal senescence and potential therapeutic options. (B-C) (B) Cadmium exposure induces cellular senescence and impairs osteogenic and adipogenic homeostasis in primary BMSCs. Primary BMMSCs were cultured in the presence or absence of 10 μM Cd for 24 h. Cellular senescence was detected by β-galactosidase (SA-β-Gal) staining, and cell proliferative capacity was analyzed by EdU staining. Scale bar = 50 μm. (C) Cd exposure increased SASP-related cytokine production and activated the NF-κB pathway in BMSCs. BMSCs were cultured for 3 hr with or without Cd exposure, and qPCR was performed to detect the gene expression levels of several SASP markers (IL-1α, IL-1β, TGF-β, CXCL-1, and VEGF). Adapted with permission from 21, copyright 2021. (D) Vessel formation was promoted by drug intervention, as shown by immunofluorescence. (E) Effects of BSTLD on angiogenesis and osteoclast activation in the epiphysis of OVX rats and immunostaining for vascular endothelial growth factor A (VEGF-A) and calcitonin receptor (CALCR) in the femoral epiphysis at twelve weeks postsurgery. Adapted with permission from 76, copyright 2022. Created with BioRender.com.

Previous studies have demonstrated that glucocorticoids inhibit osteoblast differentiation and promote osteoclast function, thereby contributing to osteoporosis 51. Additionally, glucocorticoids indirectly influence bone metabolism by regulating hormone levels. Xiaonan Liu *et al.* induced senescence in a small population of bone marrow adipocytes (BMAds) in adult mice by treating them with glucocorticoids 72. These senescent adipocytes secrete SASP factors, leading to the spread of aging to surrounding bone and bone marrow cells. The accumulation of aging cells in the local environment results in the release of inflammatory factors and other substances, ultimately causing osteoporosis. This finding provides insights into the specific mechanism by which glucocorticoids impact bone metabolism and the role of aging in bone metabolism and osteoporosis (**Figure 5**A-C).

Antianxiety treatment for cellular aging has been investigated to a certain extent. Xiaotao Xing *et al.* developed a bone-targeted delivery system for anti-aging drugs that effectively eliminated aging BMSCs 73. This treatment significantly reduced the expression of aging markers in an aging BMSC model. Additionally, cell mitosis and calcification staining experiments confirmed its ability to enhance the proliferation and osteogenic differentiation of aging BMSCs *in vitro*. This demonstrates that clearance therapy targeting aging cells can restore bone mass and bone microstructure, making it an effective treatment method for elderly patients with osteoporosis.

### 2.10 Local bone tissue vascular destruction and reduced regeneration

Changes in blood vessels within the pathological microenvironment of osteoporosis patients are observed, primarily in terms of their quantity and quality 74. The number of blood vessels in the bone tissue of patients with osteoporosis decreases, resulting in impaired blood supply and nutrient transport in the bone tissue (**Figure** 5D-E). This decrease in blood vessel quantity may be attributed to decreased angiogenesis and increased vascular destruction, possibly due to abnormal expression or insufficient release of angiogenic factors such as vascular endothelial growth factor (VEGF) 75. Furthermore, structural and functional abnormalities also contribute to the reduced quality of blood vessels in osteoporosis patients. These abnormalities include weak vascular walls, vulnerability to rupture, and diminished blood flow due to vascular dilation. The limited blood flow within the bone tissue is likely responsible for these structural changes. Functional abnormalities mainly affect vascular endothelial cells, leading to impaired endothelial function and an increased inflammatory response, ultimately resulting in blood flow disorders and decreased vascular diastolic function in osteoporosis patients 72. An inadequate blood and nutrient supply to bone tissue due to these pathological microenvironment changes can significantly hinder bone regeneration and integration, ultimately leading to bone surgery failure. Therefore, a thorough understanding of vascular changes and underlying principles within the pathological microenvironment of osteoporosis is crucial for effective treatment.

### 2.11 Bone marrow fat accumulation

**Figure 6:**
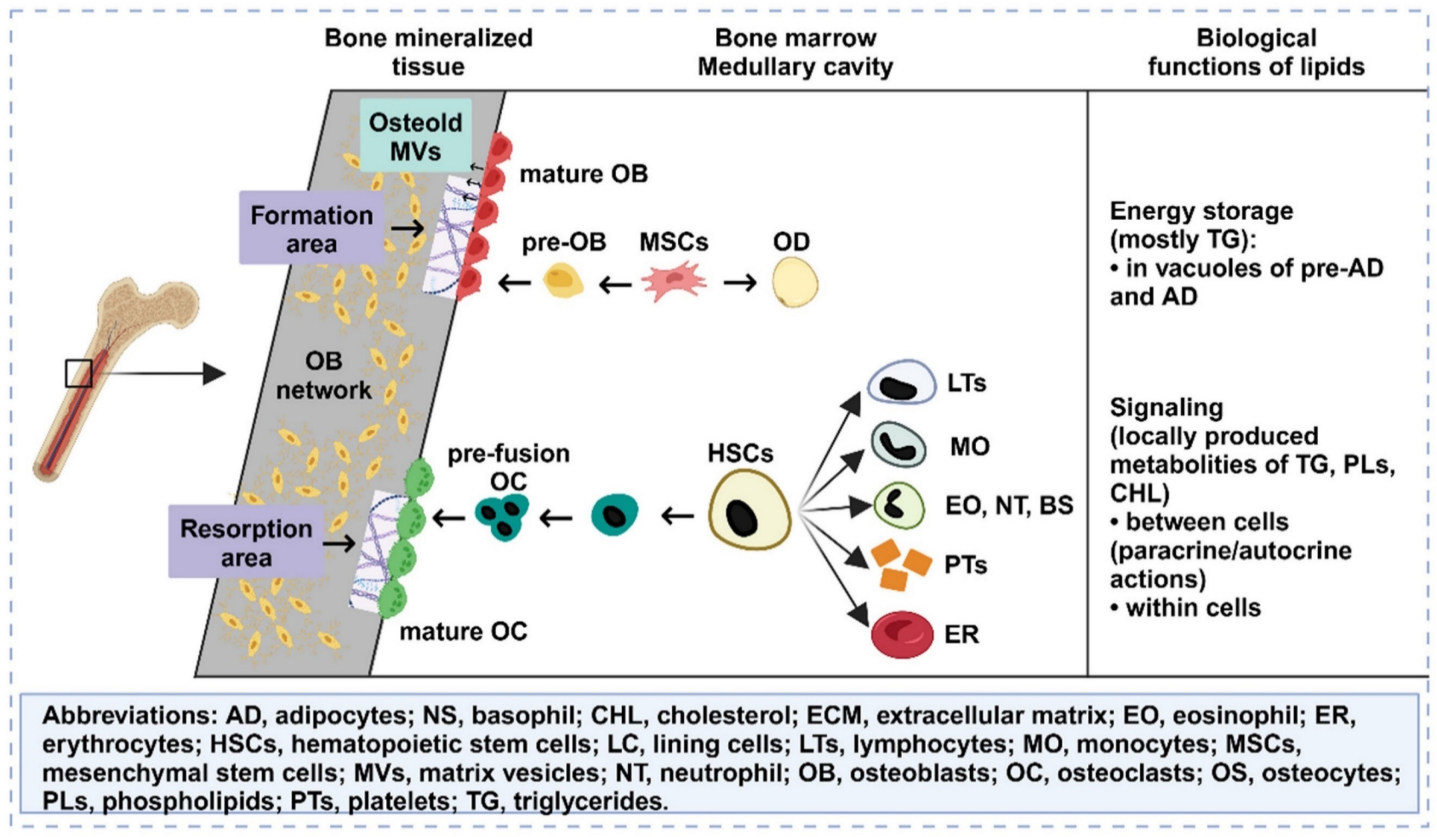
Lipids accumulate in the bone marrow, influence osteogenesis and osteoblastogenesis, and have multiple functions. Created with BioRender.com.

Osteoporosis is characterized by a decrease in BMD and an accumulation of bone marrow adipose tissue, indicating dysfunction in the affected bone tissue. However, there is ongoing controversy regarding whether the accumulation of bone marrow fat is an active or passive process. Various hypotheses propose that this could be due to an increased tendency of normal stem cells in the bone marrow to differentiate into adipocytes or a transfer of adipose tissue from outside the bone marrow to the interior 77. This may also be associated with the use of current osteoporosis drug therapies. Normal bone marrow adipocytes not only fill the expanded trabecular space but also provide necessary energy and maintain a stable microenvironment 78. However, recent studies have revealed that bone marrow adipocytes can release proteins, cytokines, and fatty acids (FAs) through autocrine and paracrine functions. Moreover, FAs in the bone marrow microenvironment can regulate the growth, differentiation, and development of bone cells through various signaling pathways (**Figure** 6). FAs can also activate/express nuclear transcription factors that are critical for maintaining bone homeostasis and influence the survival and function of neighboring OBs 79. Ultimately, this may lead to the development of osteoporosis. Given the unclear classification and functions of FAs in bone fat, further investigation into bone marrow FAs is essential. In different species, including humans, bone marrow lipid extracts consist mainly of triacylglycerols, with small proportions of phospholipids and free cholesterol 80. Adipocytes primarily derive FAs from triacylglycerols. The composition of bone marrow FAs varies more according to species than according to anatomical location. For instance, in rats, palmitic, stearic, and oleic acids were the predominant FAs, whereas palmitic, oleic, and linoleic acids were the major FAs in guinea pigs, rabbits, and human bone marrow.

FAs and their metabolites can exert regulatory effects on bone metabolism through various mechanisms, including oxidative stress, inflammation, autophagy, and apoptosis. The receptors involved in the regulation of FA-dependent bone metabolism include G protein-coupled receptors, peroxisome proliferator-activated receptors, and Toll-like receptors 81. By modulating the RANK/RANKL/OPG signaling pathway, a range of FAs can participate in the process of bone remodeling, which is closely associated with postmenopausal osteoporosis. Notably, palmitate, a long-chain SFA, is a common FA found in the bone marrow of osteoporosis patients, suggesting its potential role in the pathogenesis of this condition. SFAs can generate ROS via nonspecific peroxidation, wherein low levels of ROS regulate the cellular peroxidative state, whereas excessive aggregation induces cellular lipotoxicity 82.

Furthermore, elevated levels of free SFAs promote ROS production through mitochondrial beta-oxidation, thereby activating signaling pathways associated with apoptosis. *In vitro* and *in vivo* experimental studies have provided evidence of the negative effects of SFAs on OBs. Coculturing OBs with adipocytes leads to decreased proliferation, differentiation, and function of OBs due to lipolysis and increased levels of free FAs within the culture medium. Direct treatment of OBs with SFAs also results in lipotoxic effects mediated by oxidative stress, with adipocytes generating significant amounts of ROS and products of lipid peroxidation. However, certain FAs, such as oleic acid, have been shown to exert positive effects on osteoporosis repair in addition to their inhibitory effects 83. Oleic acid antagonizes various proinflammatory pathways activated by palmitic acid, thereby counteracting its cytotoxic effects and promoting osteoclast differentiation. The detoxifying effect of oleic acid may occur through the stimulation of palmitic acid esterification into triacylglycerols. Moreover, palmitoleic acid inhibits osteoclast formation induced by RANKL and promotes the apoptosis of mature osteoclasts. In animal models, feeding male rats virgin olive oil enriched with MUFAs prevented age-related decreases in BMD compared to feeding sunflower oil enriched with omega-6 PUFAs. By judiciously utilizing different types of FAs, it is possible to develop safer and more effective therapeutic strategies for osteoporosis treatment.

### 2.12 Genomic changes

These genes play critical roles in the development of OP (**Table** 1), and different genes are implicated in the pathogenesis of OP through different pathways. The p53 gene has the highest mutation frequency across all cancer types, and the inactivation of p53 plays a pivotal role in tumor formation 84. As a key transcription factor, p53 is directly or indirectly involved in the regulation of numerous genes responsible for responding to various stress signals, including DNA repair, apoptosis, aging, and metabolism. OP is a multifactorial disease, and a multitude of genes and signaling pathways are involved in its pathogenesis. Bioinformatics analysis of OP-related genes revealed that the correlation between the p53 gene and OP was the strongest, suggesting that p53 may play a central role in the development of OP 21. OBs originate from bone marrow BMSCs, primarily differentiating into OBs through the Wnt/β-catenin and bone morphogenetic protein (BMP) pathways and subsequently maturing into OBs under the influence of the osteoblastic transcription factors (OTFs) Runx2, Osterix, and BMP. Runx2, Osterix (Osx), and the homologous transcription factor DLX5 activate these genes, promoting further metastasis 85. The regulation of osteoblast differentiation by p53 is facilitated through miRNAs. miRNAs are small non-protein-coding RNAs that serve as key posttranscriptional regulators of gene expression. p53 can regulate the expression of several miRNAs, with miR-34 family members being the most prevalent p53-induced miRNAs. Specifically, miR-34b/c is commonly induced by p53, which inhibits Cyclin D1, CDK4, and CDK6 to suppress osteoblast proliferation and terminal differentiation via SATB2 inhibition. Dysregulation of bone metabolism-related signaling pathways leads to abnormal proliferation and differentiation of OBs and osteoclasts, resulting in a reduction in BMD and the development of OP 86. Furthermore, p53 influences bone remodeling through its regulation of bone metabolism-related signaling pathways, with the Wnt/β-catenin, OPG/RANKL/RANK, and BMP/Smad signaling pathways emerging as three key regulators of bone metabolism.

Inflammatory factors play a significant role in bone metabolism. OP is characterized by a decrease in immune system activation and increased production of inflammatory mediators due to aging and decreasing estrogen levels 87. The release of proinflammatory factors in the inflammatory microenvironment disrupts the balance of bone metabolism and contributes to the development of OP. Elevated IL-1, IL-6, IL-8, and TNF-α promote bone resorption through various mechanisms but enhance the NF-κB, RANKL/RANK and BMP/Smad signaling pathways. RANKL, a key factor in bone metabolism, induces osteoclast differentiation, activation, and survival but inhibits osteoblast survival 88. Normally, p53 acts as an inhibitor of the inflammatory response. However, loss of function or mutation of p53 leads to excessive inflammation and activation of IL-6, IL-8, and TNF-α expression. Consequently, the loss of the inhibitory effect of p53 on the inflammatory response results in the release of excessive proinflammatory factors, disrupts bone metabolism, promotes bone resorption, and decreases bone formation, ultimately leading to the development of OP.

Iron-induced cell death, known as iron death, is a programmed cell death process dependent on iron. Iron overload and the accumulation of ROS are notable characteristics of iron-related death and are significant risk factors for OP development 89. In OBs, iron overload and ROS accumulation induce iron death and impair osteogenic differentiation and mineralization, thereby compromising osteoblast function and disturbing bone remodeling. Additionally, iron-related death is exacerbated within an inflammatory microenvironment, such as in rheumatoid arthritis, where IL-6 increases intracellular iron levels, reduces ferritin expression, and triggers iron-related death in synovial fibroblasts 90. Subsequently, iron-related death leads to the release of numerous inflammatory factors. Under normal conditions, glutathione peroxidase 4 (GPX4) mitigates the inflammatory response and inhibits inflammatory cytokines by downregulating the TNF-α-mediated NF-κB signaling pathway. The supernatant of GPX4-inactivated intestinal epithelial cells contains various inflammatory cytokines, including IL-6 and TNF-α. The pathogenesis of osteoporosis has been the subject of extensive research. However, it is equally important to investigate the interactions between osteoporosis and other diseases as well as the microenvironmental changes caused by osteoporosis. This parallel exploration is crucial for promptly enhancing patients' conditions and formulating personalized medical treatment strategies with substantial therapeutic effects. In addition, twelve therapeutic targets for osteoporosis can be identified using various treatment approaches, employing combinations of different stimuli to attain more adaptable therapeutic strategies.

While the pathogenesis of osteoporosis has always been a hot topic of research, the interactions between osteoporosis and other diseases as well as the microenvironmental changes brought about by osteoporosis should also be given the same attention, which is beneficial for the timely improvement of the patient's condition and the development of personalized medical treatment strategies with significant therapeutic effects. The twelve osteoporosis therapeutic targets can also be treated with different therapeutic approaches, utilizing combinations of the efficacy of different types of stimuli to achieve more flexible therapeutic strategies.

## 3. Biophysical stimulation for osteoporotic osseointegration and osteoregeneration

### 3.1 Endogenous biophysical stimulations

#### 3.1.1 Stiffness and elastic modulus

**Figure 7:**
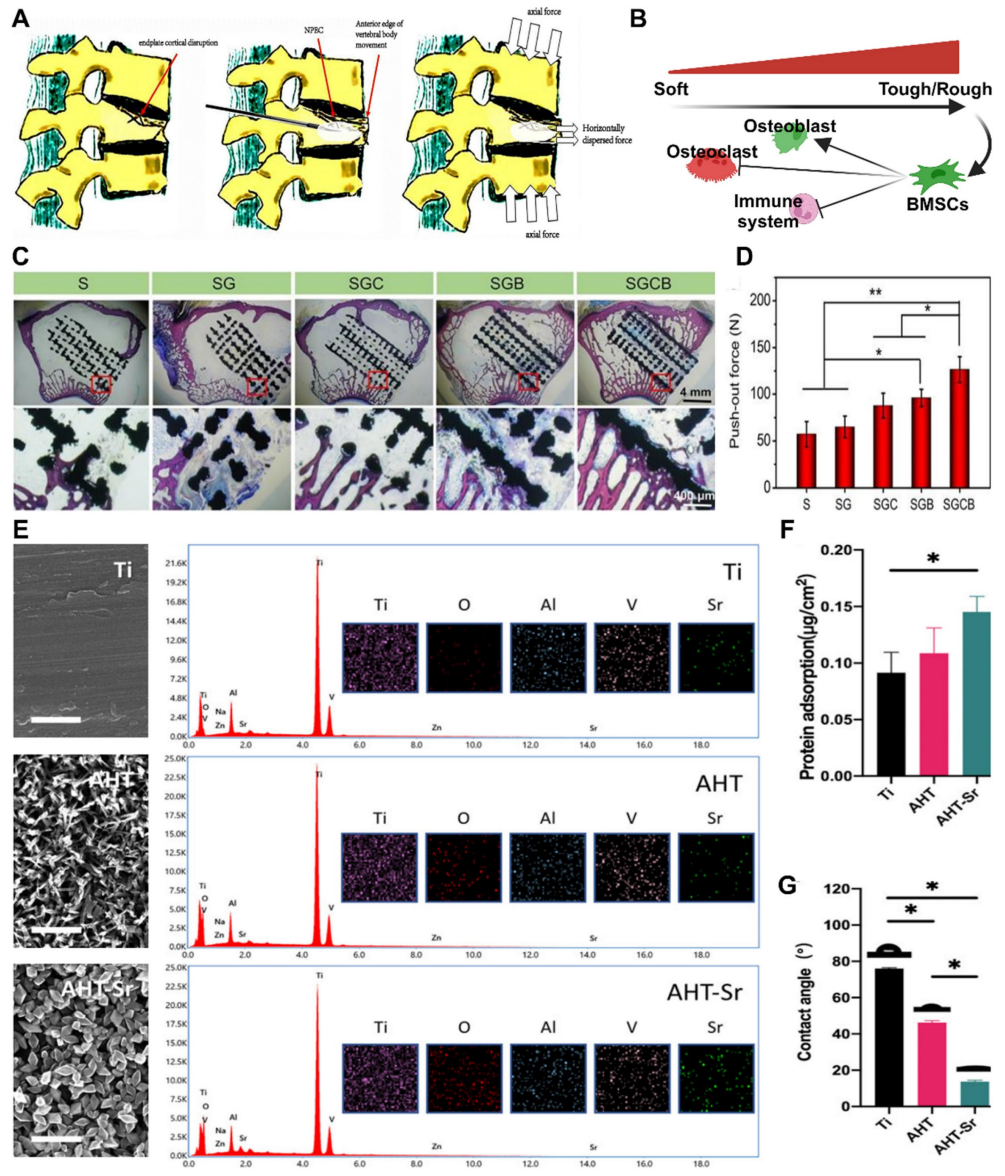
Stiffness, modulus of elasticity and surface roughness. (A) Schematic representation of refracture due to endplate cortical disruption. The presence of endplate cortical disruption is shown. Adapted with permission from 95, copyright 2021. (B) Effect of material surface stiffness on stem cell differentiation tendency. (C-D) (C) Representative histologic photographs of Van-Gieson-stained bone defect areas (black areas represent titanium alloy, red areas represent bone). (D) Osseointegration was assessed by tensile biomechanical testing 3 months after implantation (*p < 0.05, **p < 0.01). Adapted with permission from 94, copyright 2020. (E) Surface characterization of Ti, AHT and AHT-Sr surfaces, including representative SEM images (scale bar: 500 nm), EDS spectra and mapping. (F, G) Surface physicochemical properties of Ti, AHT and AHT-Sr, including the water contact angle (n = 3) and protein adsorption test results for various specimens. Adapted with permission from 96, copyright 2022.

The mechanical properties of materials have a significant effect on the behavior of bone cells. An appropriate stiffness can provide mechanical stimulation, activate cellular signaling pathways, and influence cell proliferation, differentiation, and matrix synthesis. The elastic modulus of a material is an important parameter for its mechanical properties and reflects its ability to deform under stress. In mechanics, the elastic modulus is commonly used to measure the stiffness of a material. The higher the elastic modulus of a material is, the greater its stiffness. When evaluating the suitability of a material's elastic modulus, it is necessary to compare it with that of bone tissue in the treatment environment 91. A material with a high elastic modulus and a higher elastic modulus than the surrounding cancellous bone can potentially lead to stress shielding (**Figure 7**A-D), which may result in secondary fractures of the bone tissue at the site of implantation. Therefore, in clinical applications, the elastic modulus of the material is typically reduced to enhance osteoporotic bone regeneration and bone integration. Many excellent basic materials currently have the drawback of a high elastic modulus. Extensive research has been conducted, and various technologies have been developed to decrease the elastic modulus of materials and enhance their biological activity.

Increasing the strength of materials and optimizing their structure can gradually reduce their elastic modulus. One common technique is to incorporate fillers, such as materials with porous structures or microspheres, into the base material 92. In clinical practice, polymethyl methacrylate (PMMA) is widely used as a bone cement due to its excellent plasticity and degradation ability. However, its high elastic modulus limits its application. Miao X *et al.* proposed the use of porcine small intestinal submucosa (SIS) for the production of polymethylmethacrylate (PMMA), which is a partially biodegradable mSIS-PMMA bone cement, by preparing mSIS using an *in vitro* biomimetic mineralization technique and using it as an active filler in PMMA bone cement 93. The elastic modulus of the composite bone cement is closer to that of human cancellous bone (50-800 MPa) compared to that of pure PMMA bone cement. MSC experiments showed that mSIS-PMMA enhanced cell adhesion, proliferation, and osteoblast differentiation in BMSCs (**Figure** 7D-E). Animal experiments on osteoporotic rabbits demonstrated that the mSIS-PMMA group had significantly better bone regeneration performance than the control group, as evidenced by morphological evaluation and staining. Microscopic examination revealed infiltration of fresh host bone tissue in the mSIS-PMMA group. Compared with that of pure PMMA, the degradation of mSIS-PMMA in the physiological environment promoted bone integration. Another common technique to reduce the elastic modulus is through the use of multilevel, porous, or multilayer structural designs, which alter the shape and distribution of the material. Titanium alloys, the most commonly used material for orthopedic implants, have wide application prospects due to their high mechanical strength and corrosion resistance. However, their high stiffness makes them susceptible to stress shielding-induced osteolysis and fractures. Bai H *et al.* prepared porous titanium alloy scaffolds using 3D printing technology 94. These scaffolds matched the mechanical properties of bone tissue and were loaded with BMSCs and BMP-2 double supramolecular hydrogels as bioactive composite scaffolds, which enhanced bone regeneration and integration in osteoporotic bone defects. *In vivo* and *in vitro* experiments have demonstrated that composite materials can induce the attachment of BMSCs and promote osteogenic differentiation.

However, there are still unanswered questions regarding the effects of stiffness and the elastic modulus on bone regeneration and integration in individuals with osteoporosis. First, the manuscript discusses the mechanism by which stiffness and the elastic modulus influence the behavior of bone tissue cells. Further research is needed to understand how stiffness affects the proliferation, differentiation, and synthesis of the bone matrix in bone cells. Additionally, a more comprehensive understanding of the regulatory mechanisms of cell signaling pathways and gene expression is required. Second, it is essential to conduct in-depth research on the long-term effects of stiffness and the elastic modulus on osteoporotic bone regeneration and integration. This research should focus on understanding the long-term biocompatibility of materials with surrounding bone tissue and evaluating the long-term function and integration of the repair area. This approach has significant value in guiding clinical practice and material design.

#### 3.1.2 Surface roughness

Surface roughness plays a crucial role in osteoporotic bone regeneration and integration 18. It can increase the surface area and the contact area, significantly impacting these processes. Greater surface roughness provides a larger surface area and richer microstructure, which enhances cell attachment sites, particularly for functional cells such as vascular endothelial cells 97. This is beneficial for promoting blood vessel regeneration in areas of bone defects and improving bone integration. A larger cell response area also aids in the differentiation and tissue regeneration of osteoporotic bone cells 36. Studies have shown that cell perception of surface roughness affects the activation of signaling pathways, thus influencing cell differentiation. This includes the impact on autophagy in bone cells, which offers potential new therapeutic targets for degenerative bone diseases such as osteoporosis.

In the field of clinical research, it is often necessary to enhance the surface roughness of materials. Currently, several production technologies are available for creating an ideal surface morphology, including sandblasting, acid alkali corrosion, oxidation treatment, electrochemical treatment, surface coating, ion beam engraving, excitation treatment, and 3D printing. Szurkowska *et al.* modified hydroxyapatite (HA) powder by introducing magnesium and silicon ions, and they prepared a composite bead that could transport raloxifene via a crosslinking reaction between magnetic beads and a suspension 98. The dry composite bead exhibited a rough surface under an optical microscope (**Figure** 7E-G), and SEM images also revealed a uniform and rough surface morphology. This characteristic is advantageous for promoting the regeneration of damaged bone tissue and facilitating bone integration. Geng *et al.* developed a strontium-doped coating (AHT Sr) on the surface of titanium implants through an alkaline thermal reaction 96. This coating significantly enhances the osteogenic differentiation and bone integration of BMSCs in the pathological microenvironment of osteoporosis. Researchers have evaluated the hydrophilicity/hydrophobicity of multiple samples using water contact angle measurements, and the results showed that AHT-Sr exhibited greater hydrophilicity than did pure titanium or AHT alone. This difference can be attributed to the increased surface roughness of the composite materials, and changes in surface roughness and wettability theoretically affect bone integration. In cell experiments, researchers isolated and cultured BMSCs from osteoporotic rats and evaluated the biological characteristics of the cells after AHT-Sr treatment. The results demonstrated enhanced cell attachment and migration on the AHT-Sr surface, as well as improved osteogenic differentiation. In an animal model of osteoporosis, the volume of newly formed bone and the thickness and quantity of trabecular bone surrounding the AHT-Sr implant were significantly greater than those surrounding the AHT implant. This finding illustrates the superior bone regeneration capacity of the composite materials.

Currently, the study of material surface roughness presents numerous research prospects and challenges. Further investigation is necessary to elucidate the molecular mechanisms underlying the effect of surface roughness on bone cell adhesion, proliferation, and differentiation. Additionally, there is still room for improvement and optimization in the existing surface treatment technologies. Photoelectrochemical reactions may offer additional options for regenerating and integrating osteoporotic bone.

#### 3.1.3 Surface two-dimensional micro/nanostructure

Surface two-dimensional micro/nanostructures differ from surface roughness because they focus on deliberate modifications of surface morphology and material arrangement. In this regard, they are categorized as biophysical stimuli separate from surface roughness. These structures can be further categorized into micro/nanofiber structures and texture structures, each having a unique effect on osteoporotic bone regeneration and bone integration.

**Figure 8:**
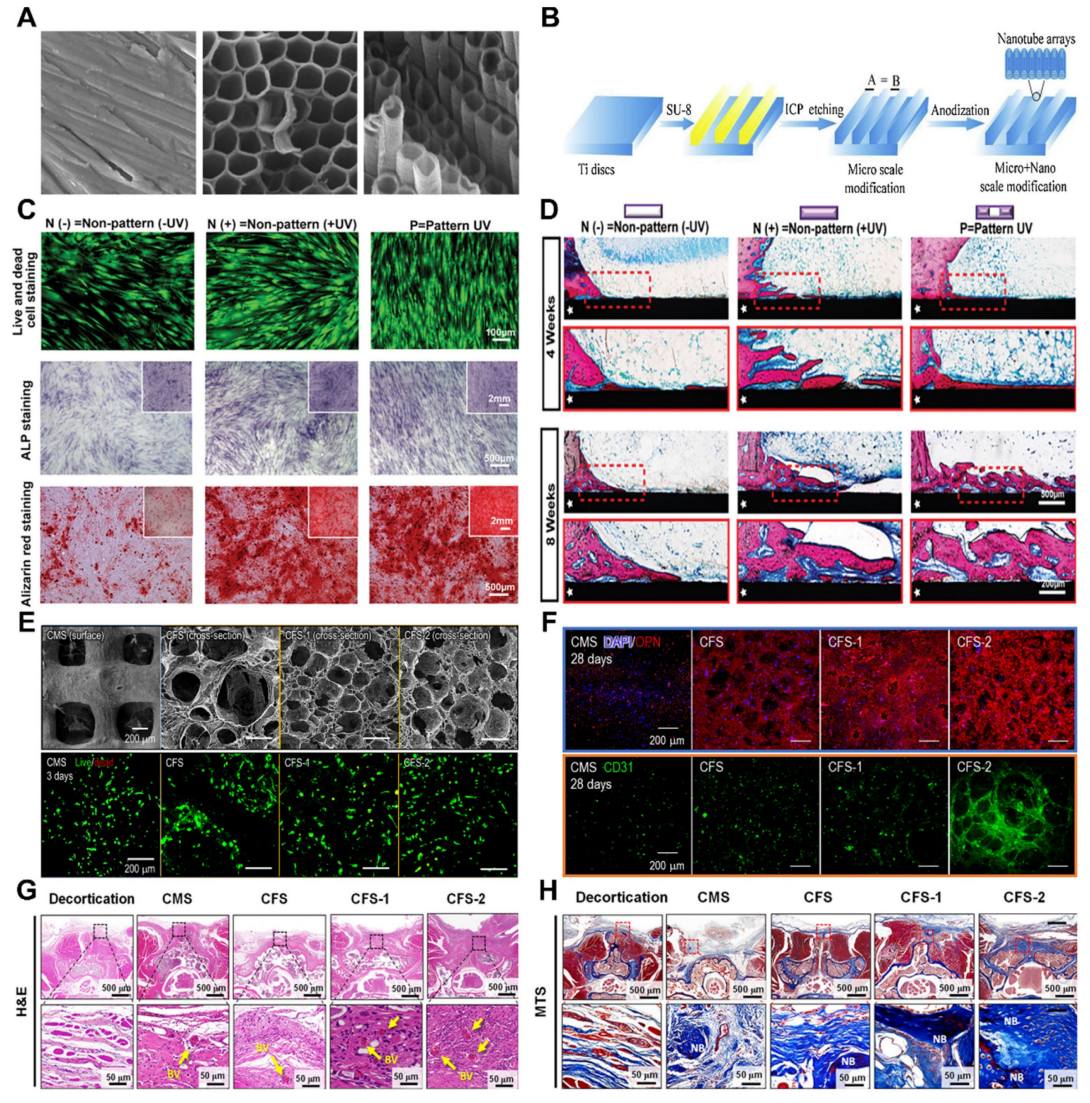
Two or three dimensions of the surface. (A) FE-SEM image of Ti and TiO2-NT arrays constructed on Ti by electrochemical anodizing. Adapted with permission from 100, copyright 2019. (B) Schematic diagram of the processes of inductively coupled plasma (ICP) etching and anodizing for the preparation of microscale grooves and nanotubes on the surface of titanium (Ti), respectively. Adapted with permission from 103, copyright 2019. (C) Osteogenic differentiation of hMSCs was promoted on the UV-patterned surface. (D) *In vivo* osseointegration was enhanced on the UV-patterned surface. Representative methylene blue images of hard tissue sections at four and eight weeks postsurgery; the area indicated by the white pentagram is the TiO_2_ matrix implant, and the red dashed box is the magnified area. Adapted with permission from 101, copyright 2023. (E-H) (E) Characterization of collagen-based cell-loaded porous constructs (CMS, CFS, CFS-1, and CFS-2) via live/dead cell assays and DAPI/ghost pen cyclic peptide staining. (F) Crosstalk-induced osteogenesis and angiogenesis between hASCs and ECs in porous cell constructs. Immunofluorescence images stained with an OPN antibody and stained with a CD31 antibody after two weeks of culture. (G) Histological analysis six weeks after implantation. Histological images showing hematoxylin and eosin (H&E) staining and cross-sections of the spinal fusion after Masson trichrome staining in the exfoliated, CMS, CFS, CFS-1, and CFS-2 groups. Adapted with permission from 104, copyright 2022.

The micro/nanofiber structure involves the creation of micron-scale fiber structures on material surfaces. These structures mimic the fibrous composition found in bone tissue, providing mechanical support and a biomimetic environment for osteogenesis. Additionally, they facilitate the attachment, proliferation, and repair of bone defects 99. For instance, titanium alloy, commonly used in clinical bone implantation treatment, often leads to low bone integration and surgical failure due to bacterial infection or metal corrosion. Implementing fibrous structural materials on the surface of titanium-based materials can greatly enhance biocompatibility, thermal and chemical stability, surface chemistry control, size control, and a high surface-to-volume ratio. Furthermore, integrating titanium oxide layers with new strategies for controlling drug transport and biodegradable metal polymers shows immense promise in developing these materials. In cell experiments, Fathi M *et al.* demonstrated the favorable effect of TiO_2_-NT fiber structures on cell attachment and growth 100. Moreover, TiO_2_-NT fibers exhibited excellent characteristics in vancomycin release tests and displayed strong antibacterial properties, providing evidence of the positive effect of the TiO_2_-NT fiber structure on osteoporotic bone integration. Common preparation methods for surface micro/nanofiber structures include anodic oxidation, electrostatic electrospinning, and ion spraying. Fathi M *et al.* employed positive oxidation to construct vertically and uniformly distributed TiO_2_ NT fiber structures on titanium sheets. The FE-SEM cross-sectional images showed vertically ordered and compact nanofiber structures throughout the entire formation, with a thickness of 40 μm (**Figure** 8A-B).

The micro/nanotexture structure refers to the formation of micron-scale texture structures or specific patterns on the surface of a material. These structures can increase surface roughness and fine structures, enhancing the mechanical anchoring and interaction between the material and surrounding bone tissue. Natural tissues often have specific surface structures for specialized functions. Current research aims to generate various surface patterns, such as stripes, grid lines, circles, squares, and stars, to construct biomimetic bone, periosteum, and muscle tissues. Research has focused on understanding how physical signals regulate cell fate, with geometric clues shown to manipulate cell behavior and fate by altering cell adhesion and morphology, as well as regulating intracellular signal transduction. Hence, constructing a physical microenvironment with surface geometric patterns on biomaterials is considered a potential tissue repair strategy. For example, micropattern surfaces with larger circumferences and relatively high aspect ratios promote osteogenic differentiation of human mesenchymal stem cells (hMSCs), whereas opposite conditions induce adipose differentiation.

Yiting Lou *et al.* designed a micropattern material based on the light response characteristics of titanium surfaces 101. After UV micropattern printing, BSA protein and hMSC micropatterns were obtained on the nanorod surface of the titanium sheet, which matched well with the designed photomask 102. *In vitro* cell experiments demonstrated that linear micropatterns and UV functionalization can enhance the adhesion and proliferation of hMSCs (**Figure** 8C-D). In animal experiments on osteoporosis models, the experimental group showed greater bone formation and bone implant contact rates than did the control group. This reveals the feasibility of applying light micropattern printing on the surface of the implant to enhance bone bonding. Zhou P *et al.* used photolithography technology to prepare materials with different widths (50 μm, 20 μm, 10 μm, 5 μm, and 2 μm) and depths (3 μm and 6.8 μm) 103. Microgroove patterns were used, followed by anodic oxidation. A dual-electrode electrochemical cell was used, and titanium-based materials were used as anodes. Voltages of 17 V and 20 V were applied to prepare nanotubes with diameters of 55 nm and 85 nm on the material surface. High-quality multilevel micronanopatterns and tightly arranged nanotube arrays were successfully prepared on the Ti surface using SEM. The surface morphology and roughness of these Ti samples were further detected through AFM. The material characterization results indicate that for a height of 3.6 μm, the modification of microgrooves and nanotube arrays with a diameter of 55 nm can improve the surface hydrophilicity of Ti substrate materials. However, when the height of the microgrooves reaches 2 μm, the surface hydrophobicity of the material actually increases when the diameter of the nanotube is 85 nm. Cell-based experiments have shown that surface microgroove patterns can effectively enhance cell adhesion and accelerate cell growth, especially the proliferation of SCC cells. The material design and experimental results confirm that micro/nanopatterns with 5.3 μm deep and 6 μm wide grooves are the optimal designs for implant surfaces. However, further animal experimental evidence is needed to meet the theoretical requirements for osteoporotic bone regeneration and bone integration.

In the study of osteoporotic bone regeneration and bone integration, the potential of surface two-dimensional micro/nanostructures is significant. In the future, there is potential to explore the effects of different types of two-dimensional micro/nanostructure combinations on the behavior of bone cells. Additionally, further investigations into the regulatory mechanisms of cell signaling pathways and gene expression have been performed. Further research can focus on the regulatory effects of surface two-dimensional micro/nanostructures on bone matrix synthesis cells, such as OBs and chondrocytes, as well as the signaling communication mechanisms with cells. Moreover, it is necessary to conduct additional experiments and research on osteoporosis to establish a foundation for future studies.

##### 3.1.4 Three-dimensional pore structure

Compared to the previously mentioned surface physical stimuli, the presence of a surface pore structure enhances the material to a greater extent. The development of a three-dimensional pore structure on the material surface provides a larger pore space, which facilitates the infiltration, directional proliferation, and angiogenesis of bone cells 105. It should be noted that a three-dimensional pore structure on the surface possesses structural characteristics in the vertical direction, in contrast to a two-dimensional nanotube array. The advantages of a three-dimensional pore structure do not necessarily stem from the ordered arrangement of pores (**Figure** 8E-H). Current studies have focused on creating three-dimensional nanopores/nanotubes on implant surfaces. However, not all three-dimensional pore structures are suitable for clinical use 106. Therefore, precise control of structural modification parameters is essential. In the manufacturing of material pores, the two most crucial factors are porosity and pore size. Previous studies have demonstrated that a porosity of 66.1% indicates optimal bone integration, whereas another study suggested that a porosity of 30-40% has a positive impact on osteogenic differentiation and bone ingrowth 107. Higher porosity promotes bone ingrowth but compromises the mechanical properties of implants, such as hardness, compressive strength, and elastic modulus. Additionally, the combination of three-dimensional pore structures, such as micro porous structures combined with nanoparticles or nanofilms combined with micro/nanopores, is a current research focus 108.

Commonly utilized techniques in this field include freeze-drying and electrolytic deposition. Currently, the key focus of research involves two areas: (1) the development of micron-scale porous nanoparticles, where a porous structure is created on the material's surface and filled with nanoparticles. The porous structure facilitates a larger surface area and more cell attachment sites, whereas the nanoparticles activate cell signaling pathways and enhance the attachment and growth of bone cells. (2) Nanofilm micro/nanopores, which entail the formation of a nanofilm on the material's surface and the creation of micro pores on the film. Nanofilms offer a flat and uniform surface, whereas micro nanopores increase the surface area and the number of cell attachment sites, thereby facilitating the settlement of bone cells and the regeneration of bone tissue.

The influence of a three-dimensional pore structure on bone integration includes several aspects 104. First, the three-dimensional pore structure on the surface increases the surface available for bone cell attachment, providing adequate space for bone cell expansion and proliferation. Consequently, this accelerates the growth and diffusion of bone cells, promoting bone integration. Second, an appropriate three-dimensional pore structure assists in guiding bone cells toward the desired differentiation direction and stimulates the synthesis and deposition of the bone matrix. These factors enhance the formation of new bone tissue, ultimately promoting bone integration and regeneration.

Vascular regeneration plays a pivotal role in the process of bone integration and regeneration. The three-dimensional pore structure on the material's surface offers guidance and support for the growth and diffusion of blood vessels, thereby promoting angiogenesis and the establishment of a robust blood supply 109. A well-developed blood supply is a crucial factor in successful bone integration because it delivers sufficient oxygen, nutrients, and cellular signals to promote bone cell activity and facilitate bone tissue repair. Additionally, the three-dimensional pore structure on the material's surface increases the contact area between the material and the surrounding bone tissue, enhancing the mechanical support performance between the two materials 110. This, in turn, aids in providing stable force transmission, safeguarding the bone structure, and promoting bone integration.

The various parameters of the three-dimensional pore structure on the surface have a significant impact on the performance and effectiveness of materials. Two key scale parameters in surface pore structures are pore diameter and pore spacing 111. An appropriate pore diameter and pore spacing can provide sufficient space for bone cells to adhere and grow, as well as allowing for the growth and transportation of blood vessels. Typically, pore diameters range from 10 to 100 μm, whereas pore spacings between 100 and 500 μm are considered more suitable. The shape of the pore structure also influences bone integration and bone regeneration. Common pore shapes include spherical, rod-shaped, and sheet-shaped shapes 112. Different pore shapes can affect the attachment and arrangement of bone cells, as well as the mechanical properties and biodegradation rate of materials. The connectivity of pore structures is crucial for the growth of blood vessels and cell migration. Good pore connectivity can promote angiogenesis and cell diffusion through the pore structure. Therefore, it is important to ensure relatively good connectivity between the pores in the three-dimensional pore structure of the surface. The pore volume fraction refers to the volume proportion of pores in the three-dimensional pore structure of the surface. An appropriate pore volume fraction can provide sufficient space for the attachment and growth of bone cells and meet the regeneration needs of bone tissue 113. Usually, a pore volume fraction between 30 and 90% is considered to indicate good bone repair efficacy. Currently, there is insufficient evidence to determine whether the optimal three-dimensional pore parameters under different models are consistent. When conducting experiments for different purposes, sufficient experimental optimization and preexperiments are recommended to achieve the optimal level of osteoporotic bone integration and bone regeneration.

### 3.2 Exogenous biophysical stimulations

#### 3.2.1 Acoustic stimulation

**Figure 9:**
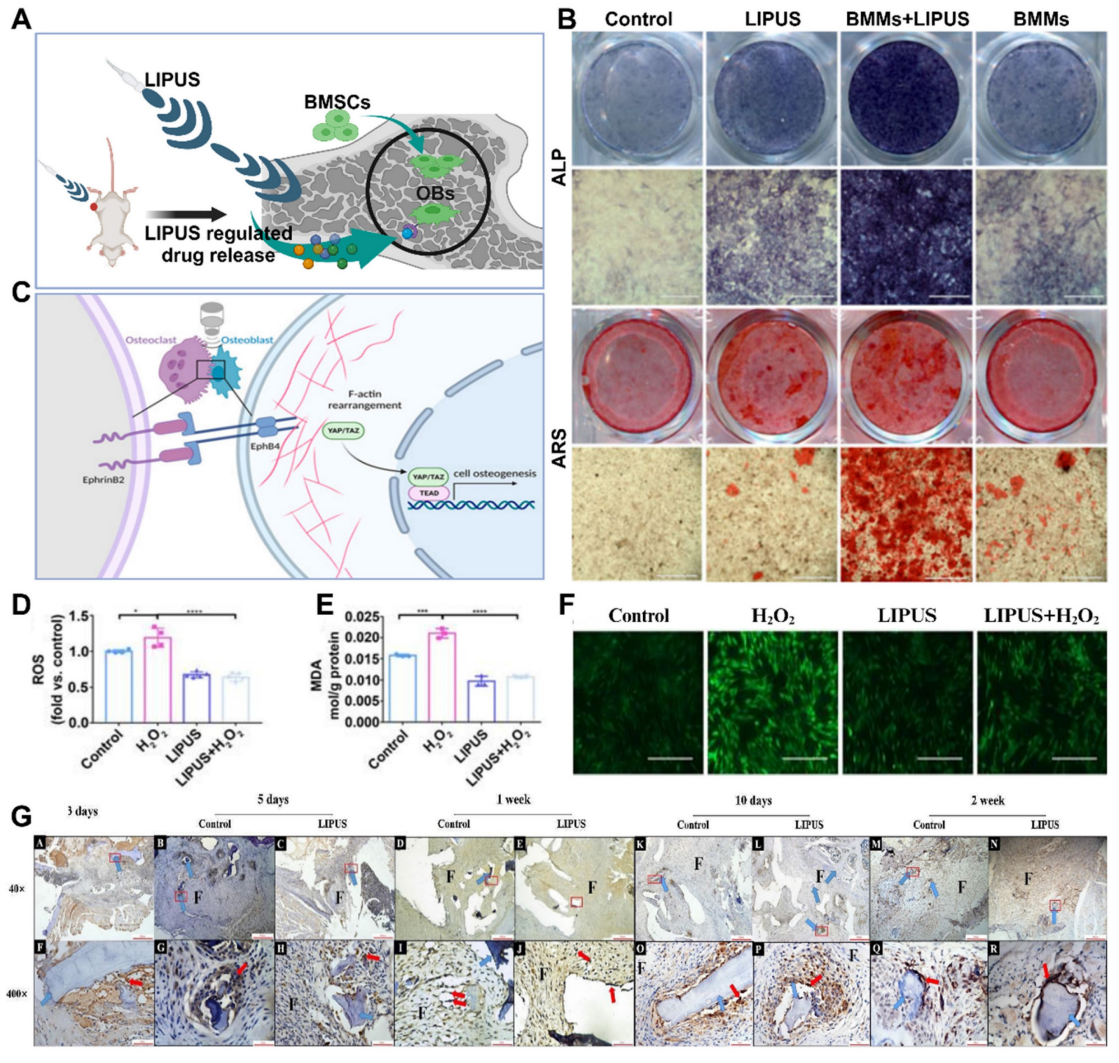
Advances in sonic stimulation in osteoporosis treatment. (A) Schematic representation of the mechanism of the effect of acoustic stimulation on cell behavior. (B) After mechanical stimulation with LIPUS, EphrinB2/EphB4 was found to be involved in regulating the migration and osteogenesis of BMSC-derived OBs in a coculture system. (C) LIPUS combined with EphrinB2-Fc-mimicked positive signaling enhances osteogenic differentiation. Adapted with permission from 115, copyright 2023. Representative images of ALP and ARS staining of each group at 7 and 21 days after osteogenic induction, respectively. LIPUS attenuates H_2_O_2_-induced oxidative stress, and intracellular ROS levels were measured by (F) DCFH-DA staining (scale bar = 400 μm) and (D) DCFH-DA fluorescence intensity. (E) The MDA content of PDLCs was measured using an MDA assay kit. Adapted with permission from 116, copyright 2020. (G) Immunohistochemical staining for Mac-2 in the control group and LIPUS group on days 3, 5, 7, 10 and 14 after surgery. Adapted with permission from 117, copyright 2019. Created with BioRender.com.

Vibratory stimulation, such as low-frequency shaking and shaking, promotes osteoblast proliferation and matrix synthesis to increase bone formation. This stimulation can be applied around bone scaffolds or implants to promote bone regeneration and osseointegration by delivering vibratory stimulation. Acoustic stimulation utilizes the vibratory action of sound waves to stimulate bone tissue. For example, the application of low-intensity pulsed acoustic stimulation (LIPUS) and high-intensity focused ultrasound stimulation (HIFU) can promote osteoblast proliferation, osteogenesis, and angiogenesis (**Figure** 9A). In recent decades, LIPUS has achieved significant results in the treatment of fractures and other bone defect diseases, and many studies have verified the promotional role of LIPUS in bone regeneration and osseointegration and explored the mechanism of the stimulatory effect in depth 114.

Changes in bone tissue are influenced by various factors, including mechanical loads, chemical signals, and bone tissue damage 118. Sound waves can serve as a source of vibratory mechanical loads, which can impact the structure and strength of bone. Stronger cortical bone is typically associated with increased mechanical loads, whereas weakened cortical bone is associated with decreased mechanical loads 115. Although the precise mechanism by which vibratory stimulation affects bone tissue remains unclear, further studies are required to provide additional evidence. Initial studies have suggested that the application of LIPUS (low-intensity pulsed ultrasound) at any stage of fracture healing can contribute to improved bone regeneration, indicating that LIPUS may have diverse functions and effects (**Figure** 9B-C). However, before mechanical loads can influence bone, they must first be translated into biochemical signals through a process called mechanotransduction. Vibratory stimuli can induce fluid flow around osteocytes, resulting in the exertion of shear stresses on these cells 119. Mechanosensory receptors then perceive these forces and convert them into biological signals.

Current research on LIPUS mechanosensing receptors has predominantly focused on the activation of integrins and kinase pathways. Integrin activation leads to the formation of structures called adhesions or focal contacts, which enable connections between the cytoskeleton and extracellular matrix. These connections play a crucial role in osseointegration and bone regeneration. Additionally, LIPUS has been found to enhance blood vessel formation 120. LIPUS increases the expression of VEGF and upregulates the levels of IL8 and IL37, which are vital signaling molecules in the angiogenic pathway. Further studies have demonstrated that LIPUS promotes tube formation in human umbilical vein endothelial cells and increases both the number and size of blood vessels. These functions are essential for facilitating material exchange during the stages of bone regeneration and osseointegration.

Other hypotheses regarding the mechanisms of LIPUS in bone tissue include the activation of calcium channels and calcium signaling, cilia activation, and β-linker protein signaling via the Wnt signaling pathway 121. Although there is evidence suggesting an association between these signaling pathways and vibratory stimulation, more comprehensive studies are needed to establish definitive evidence.

The effects of LIPUS on osteoporotic bone regeneration and osseointegration, as well as its application in related fields, have been extensively studied. These studies have primarily focused on assessing cell morphology and attachment, cell proliferation and viability, osteogenic differentiation, mineralization, bone volume, and blood vessel formation (**Figure** 9D-G). Some studies have demonstrated that LIPUS can modify cell morphology, attachment, and proliferation viability when cells are inoculated on scaffolds 122. However, other studies have concluded that LIPUS does not have an impact on cell proliferation, highlighting the need for additional research in this area.

The effect of LIPUS on osteogenic differentiation can be evaluated by measuring biochemical markers or analyzing cell morphology to assess osteogenic differentiation. The most commonly used markers for this purpose are alkaline phosphatase (ALP), an early osteogenic marker, and late osteocalcin (OCN), a marker of late osteogenesis 114. Numerous studies have indicated that the application of LIPUS increases the activity and expression of ALP or OCN, providing evidence of its osteogenic potential.

Bone mineralization aims to create hard tissues capable of withstanding mechanical loads, and LIPUS has been shown to be effective at promoting bone mineralization both *in vivo* and *in vitro*. For instance, Ying *et al.* measured calcium deposition in Ti3Al1V scaffolds implanted in New Zealand White rabbits inoculated with MC6T4-E3 cells and concluded that LIPUS increased calcium deposition by week 6 116. However, some studies have yielded contradictory results, failing to detect a significant pro-mineralization effect of LIPUS. Therefore, further extensive research is necessary to validate its efficacy.

Different LIPUS parameters have varying effects on the results of experiments, making it important to study the most effective parameters for osteoporotic bone regeneration and osseointegration in clinical applications. To determine the appropriate experimental conditions, several studies have compared different LIPUS parameters in controlled experiments. One such study compared the effects of ultrasound at 1 MHz and 3.2 MHz on MC3T3-E1 cells cultured on Ti6Al4V scaffolds *in vitro* and *in vivo*. The study revealed no significant differences between the two frequencies in terms of osteogenic differentiation, bone volume, maturation, or scaffold growth/porosity occupancy. Although the lower frequency (1 MHz) resulted in slightly higher values of ALP activity, OCN production, and pore occupancy, the difference was not statistically significant enough to be considered significant. Experimental evidence has also shown that COX-2 and PGE2 expression was greater in the 30 mW/cm^2^ ultrasound intensity group than in the 150 mW/cm^2^ group, which is the intensity approved for clinical use 119. Typically, low-intensity ultrasound is applied in a pulsed manner rather than continuously. Pulsed ultrasound has been found to be more effective than continuous ultrasound in improving cell proliferation after three days of stimulation. These results suggest that different ultrasound intensities may be better suited for promoting different aspects of bone formation. However, further studies are needed to identify the ultrasound parameters that are most effective for bone regeneration and osseointegration. Additionally, more experimental evidence of LIPUS in other pathological models is required to demonstrate its potential for widespread use.

#### 3.2.2 Electrical stimulation

**Figure 10:**
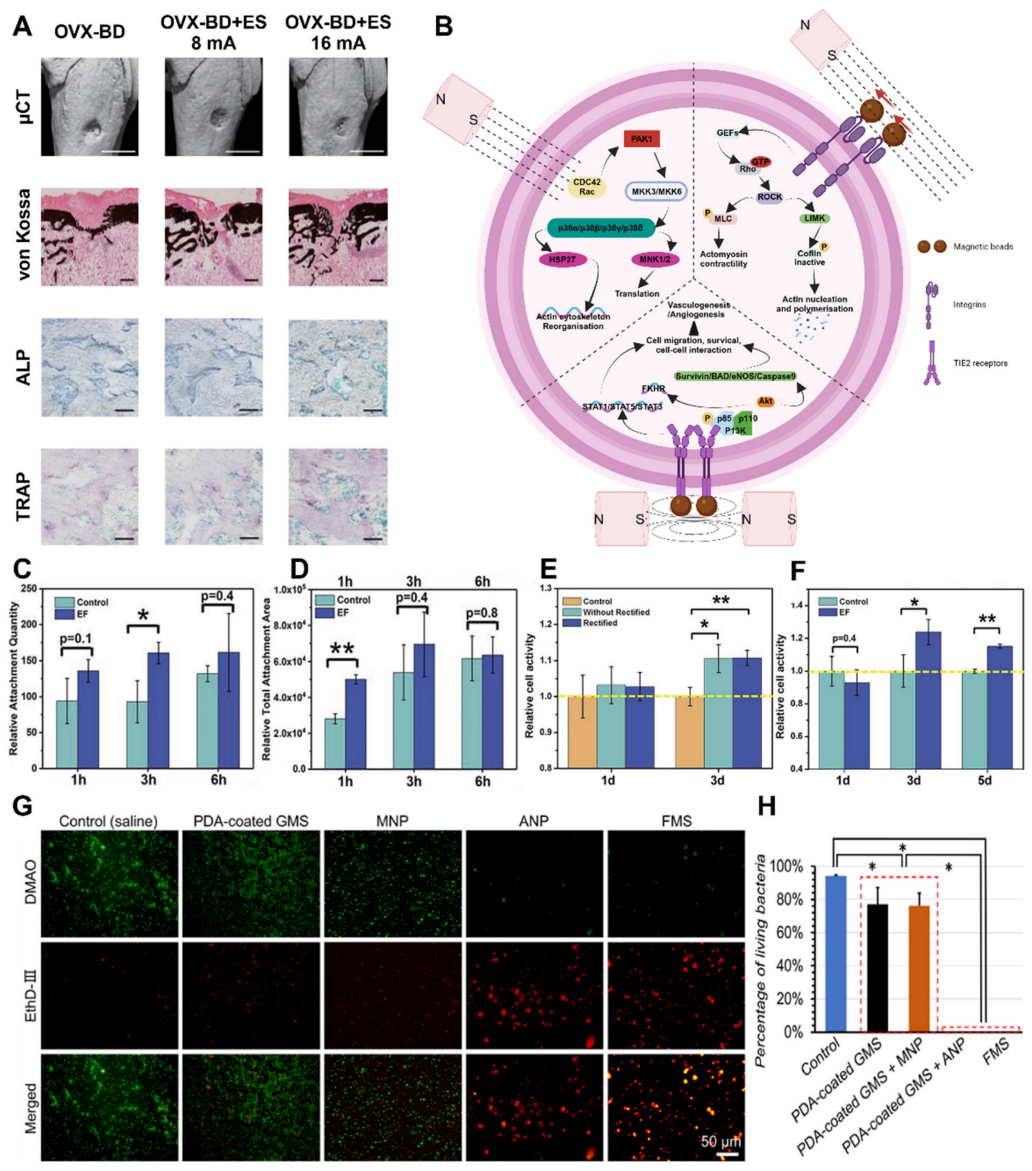
Advances in the study of electrical and magnetic stimulation in the treatment of osteoporosis. (A) Morphologic changes in epiphyseal defects in osteoporotic rats in response to electrical stimulation. Adapted with permission from 129, copyright 2021. (B) Magnetic drive-in cells and strategies used for differentiation and regeneration. (C-F) (C) Morphology and proliferation of MC3T3-E1 cells. The relative numbers of attached MC3T3-E1 cells after 1, 3, and 6 h of stimulation were 44.68%, 72.76%, and 22.22% greater than those in the no-treatment group, respectively. (*p < 0.05, n = 10). (D) Relative total attachment area of MC3T3-E1 cells after 1, 3, and 6 hours of stimulation. 78.37%, 29.05%, and 3.06% greater in the EF-treated group than in the control group (**p < 0.01, n = 10). (E) Comparison of cell proliferation between the fractionated and unfractionated groups after 1 and 3 days of stimulation. (F) MTT results showing that MC3T3-E1 cells proliferated after 1, 3, and 5 days of EF stimulation after distillation. Adapted with permission from 132, copyright 2019. The proliferation rate of the MC3T3-E1 cells was 23.82% and 15.18% greater than that of the control group after 3 d and 5 d of stimulation, respectively. Two-sample t tests were used to analyze the data, and the significance levels were ×*p < 0.05 and **p < 0.01; n = 4. (G, H) Antimicrobial properties of FMS *in vitro*. Adapted with permission from 133, copyright 2023. Created with BioRender.com.

Electrical stimulation has been shown to be a highly promising method for promoting osseointegration in bone-anchored implants. This method is commonly used in the rehabilitation of hearing and limb loss and is widely utilized in edentulous patients. Inadequate osseointegration is a major contributor to implant failure and can be prevented by accelerating or enhancing the process of osseointegration through artificial means 123. The use of electrical stimulation to promote osseointegration has been extensively studied in both *in vitro* and *in vivo* models for several decades. Various approaches have been explored, including different electrode configurations and parameters, as well as different current sources (**Figure** 10A). However, it is important to note that *in vivo* studies have only been conducted on animal models and have not yet been reported in human subjects.

The concept of electrically stimulated bone growth was first described by Fukada *et al.* in the context of enhancing osteogenesis in fracture healing 124. The authors proposed that the electric field is generated by mechanical stress applied to the bone, which compresses the tubular structure and causes fluid containing ions to flow through the tubular system, thereby promoting bone healing. Essentially, when forces are exerted on bone tissue, they generate an electrical signal due to the movement of ions, a phenomenon explained by the piezoelectric theory. Electrical stimulation has demonstrated success in various bone repair applications, such as nonhealing fractures, osteoporosis, and osteonecrosis. In these cases, electric fields can be delivered directly, indirectly (through capacitive or inductive coupling), or in combination. However, the exact mechanisms underlying this effect have not yet been identified, and the optimal stimulation parameters that yield the greatest impact are still unknown.

In applications related to osteogenesis, current is transmitted through a cathodic stainless-steel wire located near the fracture site to enhance bone formation between two segments. This process does not require an electrode-bone interface prior to stimulation. In osseointegration-related applications, the current is delivered through the implant itself to enhance the bond between the implant surface and the adjacent bone. Consequently, interference with the electrochemical properties and biological bonding between the implant and its surrounding microenvironment is a major concern (**Figure** 10C-F). Studies have primarily focused on current-controlled stimulation using DCs and pulses. Bodhak *et al.* conducted an experiment in which human fetal OBs (hFOBs) were stimulated with constant DC at 5, 15, and 25 μA for 8 minutes every 15 hours 125. After 5 days of stimulation, they observed a significant increase in the number of stimulated cells‒material interactions and viable cell density compared to nonstimulated surfaces. Additionally, they found that 25 μA stimulation was more effective than 5 and 15 μA stimulation. Kim *et al.* performed a study on rat cranial OBs (rcOBs) using biphasic pulses of 20 μA (1.5 μA/cm) with a pulse width of 32 μs and a frequency of 3000 Hz 126. They investigated two stimulation patterns: interrupted stimulation for 6 hours per day and continuous stimulation for 24 hours per day. A significant increase in cell proliferation was found after 2 days of continuous stimulation compared to interrupted stimulation and nonstimulated surfaces. Biphasic stimulation also increased vascular endothelial growth factor production but did not stimulate osteoblast differentiation.

In a recent *in vitro* study, MC3T3-E1 OBs were cultured on Ti6Al4V surfaces and stimulated continuously for 3 days in a manner similar to peripheral nerve stimulation for restoring sensory feedback in prostheses 127. The study compared different pulse amplitudes (10 and 20 μA) and frequencies (50 and 100 Hz). Other parameters include the negative pulse width (500 μs), interphase delay (50 μs), and sampling frequency (100 kSPS). were kept constant throughout the experiment. Compared with no stimulation, electrical stimulation had a positive impact on osteoblast survival, soluble collagen production, attachment, and diffusion on the Ti6Al4V surface under all stimulation conditions. Among the conditions tested, 20 μA was found to be the most favorable amplitude, although the difference compared to 10 μA was not statistically significant 128. Cell proliferation and collagen production were greater at 50 Hz than at 100 Hz and in the control. The highest osteoblast density was observed at 100 μA and 3 Hz after 20 days, showing a nearly 120% increase in cell number and a fivefold increase in collagen production compared to nonstimulated surfaces. No morphological or pH differences were detected between the stimulated specimens and controls.

Electrical stimulation applied directly through implants for the purpose of accelerating osseointegration has also been investigated *in vivo* 129. Several studies have reported promising results. For instance, in a rabbit model, Isaacson *et al.* applied an electric field with a potential difference of 0.55 V. Gold-plated Ti6Al4V implants were stimulated by direct current and placed in the femoral marrow channel 130. The anode was positioned in the adjacent muscle tissue approximately 1.5 cm from the periosteum. Stimulation was carried out for 3 or 6 weeks. Although electrical stimulation did not improve the histologic assessment of the postural bone index or mineral attachment rate, it did induce trabecular bone growth around the stimulated implant.

In another rabbit model, Buch *et al.* applied a constant direct current of 5, 20, or 50 μA for 3 weeks 131. The implant was placed between a titanium cathode and a platinum-iridium anode proximal to the tibial metaphysis. The study revealed that the bone mineral content was significantly greater in the specimens stimulated with 20 μA and 50 μA than in those stimulated with 5 μA or the control specimens. However, there were no qualitative differences observed between the stimulated and control groups, and no infections or severe inflammatory reactions were observed. Notably, a burnt black ring was observed around the anode in the 50 μA group, and an overgrowth of bone tissue at the cathode was observed in all the stimulated samples. In the Beagle model, Bins-Ely *et al.* placed commercially pure titanium grade IV implants 2 mm below the tibial crest bone. They applied constant currents of 10 and 20 μA for 7 and 15 days, respectively, using an electronic device attached to the implant attachment region. This study revealed significantly greater bone implant contact after 10 days of stimulation at 20 μA than after 15 μA or control stimulation 132. However, after 7 days, no significant change in contact with the bone implant was observed in either group. The *in vivo* studies reviewed demonstrated more consistent results than did the *in vitro* studies. However, it is important to emphasize the differences between the study designs, such as the animal model used (rabbit, beagle, dog, sheep), the location of the implant (medullary channel, tibia, mandible, femur), the metal alloy (commercially pure titanium [grades I-IV] and Ti6Al4V), the type of implant (dental implants, porous cylinders, cylinders with chambers), the experimental timetable (duration of stimulation and evaluation time points) and the applied electrical stimulation (amplitude, mode, duration, control unit) 127. Despite the differences in protocols used between *in vivo* studies, there are several recurring similarities, and both *in vitro* and *in vivo* models have shown that OBs or bone tissue are sensitive to the size of the electric field, the duration of the stimulus, the frequency of the stimulus, and the protocol used to deliver the stimulus.

#### 3.2.3 Magnetic stimulation

Magnetic forces can be easily utilized for interventions, are sustained over long distances and time periods, and have minimal direct side effects on the tissue microenvironment (**Figure** 10B). Consequently, developing a bioactive coating that can be mechanically stimulated through magnetic field control is highly suitable for meeting clinical requirements, offering several advantages over alternative methods and materials. Hydrogels, for instance, can be readily magnetized by incorporating magnetic nanoparticles due to their direct interaction with bone tissue. These hydrogels exhibit the potential to serve as magnetic actuators, as they can promptly respond to applied magnetic fields and achieve controlled changes. Previous research has demonstrated the significant osteogenic effect of magnetically actuated polycaprolactone (PCL) scaffolds on bone regeneration, with the deformation of the scaffolds induced by magnetization leading to mechanical stimulation of cells 133. However, whereas the focus of current investigations on magnetically driven hydrogels lies in the development of three-dimensional scaffolds for tissue engineering, challenges persist in generating controlled magnetic driving forces and mechanical stimulation on the surface of implants. In addition to exploring novel magnetically driven carrier materials, attention should also be given to the effects of the frequency, signal strength, and duration of magnetic stimulation on osteoporotic bone regeneration and osseointegration to determine the optimal parameters for clinical applications (**Figure** 10G-H). The two commonly employed magnetic fields in current research are static magnetic fields (SMFs) and pulsed magnetic fields (PEMFs) 134.

The SMF is a commonly used type of magnetic field in clinical practice, particularly in dentistry. Unlike PEMFs, SMFs do not require a power device, making them more convenient and easier to use. Additionally, it is widely utilized in clinical settings due to its convenience and suitability for long-term use, without causing any harmful physical irritation to the surrounding tissues, such as heat or danger from electric shock 135. Rare earth magnets that generate SMFs have been applied in various dental treatments, including magnetically fixed appliances for implants or tooth-retaining overdentures, maxillofacial prostheses after trauma and cancer surgery, and orthodontic procedures such as gap closure, molar distal intrusion, tooth traction, and palatal expansion 136. The application of SMFs has been shown to improve implant stability, reduce the risk of bone loss, and minimize bacterial contamination during the initial weeks of healing (**Figure** 10G-H).

Recent investigations have demonstrated that subjecting sandblasted, large-grit, acid-etch-treated titanium implants to a 15 millitesla(mT) SMF triggers accelerated early peri-implant bone formation and osseointegration in rabbits. However, the effect of SMFs on osteoblast differentiation and growth remains controversial. Studies have shown that exposure to SMFs does not significantly affect the proliferation of rat cranial OBs or osteoblast-like cells 137. Conversely, the proliferation of human OBs was reduced upon exposure to continuous low-intensity SMFs. Moreover, SMFs produced by corrosive currents inhibited human osteoblast differentiation. On the other hand, SMFs were found to enhance osteoblastic differentiation in rat OBs, human osteoblast-like MG63 cells, and dental pulp cells. Furthermore, a relatively low-intensity 15 mT SMF among moderate SMFs (ranging from 1 mT to 1 T) enhanced the proliferation and osteogenic differentiation of human BMSCs *in vitro*. Nevertheless, further studies on the mechanisms underlying the effects of PEMFs on BMSCs or OBs are scarce, despite evidence suggesting that PEMFs can induce early osseointegration of implants and promote bone regeneration on three-dimensional (3D) scaffolds 138. Therefore, more research is needed to explore the specific pathways through which these effects occur.

Currently, PEMFs are less commonly used in clinical practice than SMFs. However, further investigation of PEMFs is necessary to develop their applications. It is widely recognized that PEMFs can generate pulsating magnetic fields *in* living organisms, which can expedite the healing process, reduce postoperative pain, and promote faster tissue swelling ablation. Early devices using magnetic actuation were primarily tested on animals and utilized implantable or semi-invasive electrodes to deliver direct current to the site of fracture 139. More recent research has focused on depositing magnetic particles on implant surfaces to create biocompatible magnetically actuated coatings. For instance, Zhuang *et al.* prepared magnetically actuated MC coatings on titanium substrates using alternating potential-assisted electrochemical deposition (AP-ECD) of iron oxide nanoparticles (IOPs) 138. They evaluated cellular responses to mechanical stimuli generated by magnetically actuated IOP‒MC coatings with varying amounts and distributions of IOPs.

Furthermore, the mechanism underlying the enhanced magnetically driven osteogenic differentiation of MC3T3-E1 cells was confirmed. Compared with the MC group, the coating with a mass ratio of 0.67 exhibited a relatively significant mechanical stimulation effect, as evidenced by the enhanced ALP activity in the SMF group. By depositing IOPs at three different positions on the coating and maintaining a fixed mass ratio of 0.67 to modulate the mechanical stimulation, the O-IOP-MC coating showed a significantly greater ability to promote cell spreading, differentiation, and expression of osteogenesis-related genes in MC3T3-E1 cells. These effects can be attributed to the extracellular magnetically driven deformability of the coatings and the activation of intracellular magnetically driven motility and mechanotransduction signaling pathways in the IOPs 140. However, further research is needed to understand the mechanism through which magnetic stimulation alters the direction of cell differentiation, and additional material combinations should be explored for broader application purposes.

Although the precise impact of magnetic stimulation on cellular physiology is not yet fully understood, recent research has demonstrated that the regulation of physiological processes by PEMFs depends on the physiological condition of damaged tissues and the effective dosimetry of the applied PEMF at the target site 141. Consequently, numerous studies have investigated the effects of magnetic stimulation on the proliferation of OBs and have emphasized the dose responses of various factors, including signal waveforms, PEMF intensity, frequency, and duration of exposure. Different types of waveforms are found in existing PEMFs, such as asymmetric, biphasic, sinusoidal, quasirectangular, and trapezoidal waveforms. Of these, quasirectangular and quasitriangular waveforms have been approved by the FDA as safe and effective treatments for fractures. Recent studies on PEMF amplitudes have identified three amplitude windows where the highest level of cellular activity response is observed: 50-100 mT (5-10 Gauss), 15-20 mT (150-200 Gauss), and 45-50 mT (450-500 Gauss), whereas the range of 10-100 mT is associated with the maximum response. Clinically used EMFs for treatment are typically at frequencies lower than 100 Hz, with flux densities ranging from 0.1 mT to 30 mT. Most studies focused on fracture healing have provided evidence supporting the finding that an increase in the average daily dose of PEMF stimulation leads to a faster rate of fracture healing 142. For instance, a recent study that followed 1,382 patients treated with PEMF stimulation revealed that each additional hour of treatment per day reduced the average healing time by 6 days. Despite positive results from *in vivo* and *in vitro* studies, magnetic stimulation therapy requires more precise and better controlled treatment modalities. Variations in observations and results can be attributed to factors such as the utilization of different animal species, varying implantation sites, different biomaterials (e.g., ceramics or metals), and differences in intensity, frequency, signal waveforms, and stimulation duration among different studies.

#### 3.2.4 Photothermal stimulation

**Figure 11:**
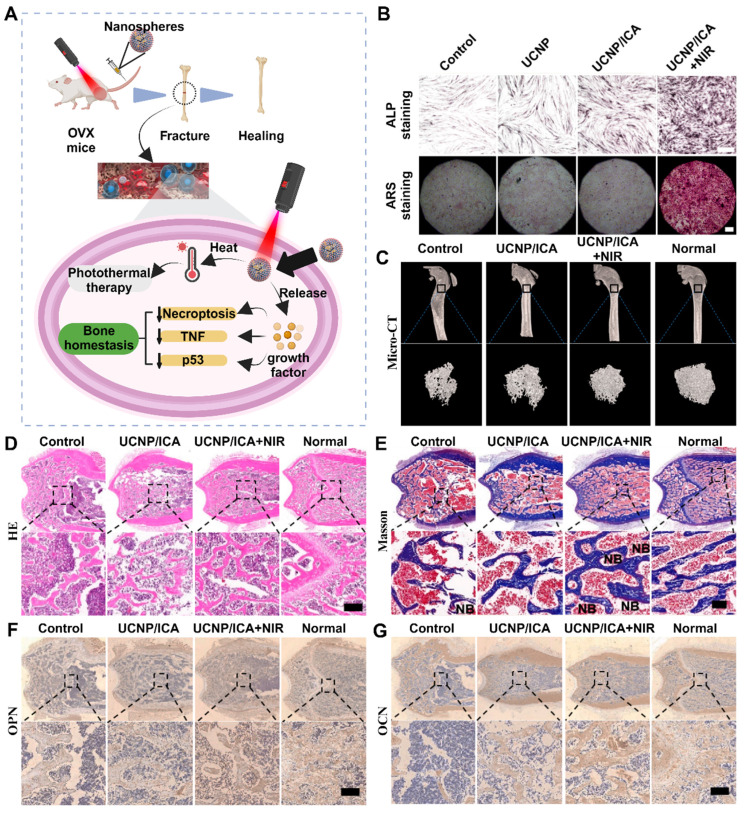
Effect of photothermal stimulation on pathological changes in osteoporosis. (A) Schematic diagram of the mechanism of action of photothermal stimulation. (B) ALP/ARS staining of osteogenically differentiated MSCs induced by different treatments. (C) Micro-CT analysis of bone indices in OP rats after different treatments. (D) Immunohistochemical staining of H&E, Masson trichrome, and femoral end sections of rats given different treatments for assessing regeneration of bone defects after eight weeks of treatment. H&E staining of femoral end sections from different treatment groups. (E) Masson trichrome staining of femoral end sections from mice given different treatments. The improvement in bone structure in the UCNP/ICA+NIR group was similar to that in the normal group and greater than that in the UCNP+ICA and UCNP/ICA groups. Note: New bone. (F) Immunohistochemical staining showing OPN and (G) OCN protein expression in terminal femoral sections from mice subjected to different treatments. Adapted with permission from 150, copyright 2022. Created with BioRender.com.

Photostimulation is the use of visible or near-infrared (NIR) light to stimulate bone tissue directly or indirectly through thermal stimulation (**Figure** 11A). Photostimulation can promote bone formation by stimulating osteoblast proliferation through the activation of photosensitive pigments or modulation of cellular metabolism. Thermal stimulation can be achieved through heat plus therapy, hot packs, cryostimulation, or temperature change cycling. Additionally, moderate photothermal effects can eliminate bacterial membranes (**Figure** 11B-C), improve blood circulation, enhance oxygen and nutrient supplies, accelerate osteoblast metabolism and synthesis, and aid in bone regeneration and osseointegration 143. Implant failure often occurs due to microbial infections, which can lead to the dislodgement of bone implants such as titanium. Current solutions rely heavily on antibiotics, but their effectiveness is limited by their narrow antimicrobial range and increasing bacterial resistance. Therefore, it is important to develop a novel strategy that can effectively eliminate bacterial membranes without promoting resistance while also enhancing bone regeneration and osseointegration at the bone-implant interface.

Photothermal stimulation is a noninvasive physical stimulation technique that has been extensively studied for its ability to eliminate biofilms. This method offers deep tissue penetration, adaptability, high selectivity, a low risk of drug resistance, and minimal side effects 144. It is also simple and easy to implement in clinical settings. Commonly used photothermal agents include metal nanoparticles, organic molecules (e.g., porphyrin, indocyanine green, and thiadiazole derivatives), carbon-based materials (e.g., graphene oxide, carbon nanotubes, and carbon nitride), and metal sulfides (e.g., copper sulfide, cuprous sulfide, and molybdenum disulfide) 145. Recent research has indicated that mild-intensity photothermal stimulation can effectively eliminate biofilms and enhance the activity of osteogenic active substances without causing excessive damage to tissue cells (**Figure** 11D-G). However, the precise mechanisms by which photothermal stimulation affects osteogenic differentiation and growth are still under investigation. Stauffer *et al.* suggested that heat treatment may accelerate bone regeneration by promoting biomineralization and upregulating the expression of ALP and heat shock protein (HSP) 146. Xue *et al.* experimentally demonstrated that heat treatment is beneficial in the middle and late stages of bone healing and promotes regeneration 147. Furthermore, the use of NIR devices for thermotherapy in photothermal stimulation has gained attention due to their portability, cost-effectiveness, and convenience compared to expensive magnetic resonance and ultrasound devices.

As a rapidly emerging therapeutic modality, NIR light-assisted thermotherapy has achieved remarkable advancements in tumor ablation and antimicrobial therapy 148. Nevertheless, it is crucial to consider that excessive temperatures during treatment can lead to damage in normal tissues at the site of the lesion. Therefore, it is necessary to explore NIR light-triggered antimicrobial therapies that can synergize within a safe temperature range.

Phase change materials (PCMs) are excellent platforms for controlled release due to their precise temperature responsiveness. By reaching the melting point of the PCM, a solid‒liquid phase change can be triggered, leading to the release of the active ingredient in conjunction with the carrier flow. Numerous FAs and fatty alcohols have melting points between 20 and 80°C. Notably, a mixture of lauric and stearic acids in a 4:1 weight ratio exhibited a single solid‒liquid phase transition temperature of 39°C, which is slightly above normal body temperature. Consequently, this combination generates a gentle and safe photothermal stimulus. The combination of NIR light and photothermite induces a temperature increase that can serve as a trigger for the solid‒liquid transition of the PCM 149. By integrating the PCM into a scaffold, the stimulation and controlled release of antimicrobial agents can be achieved through laser irradiation. In this study, PCL nanofibers were identified as promising candidates for use as drug carriers and photothermal agents. PCL nanofibers are two-dimensional (2D) layered materials that possess excellent biocompatibility, superior photothermal conversion efficiency, and a large specific surface area.

Bisphosphonate (BP) exhibits appealing potential for various biomedical applications, such as tumor ablation, bioimaging, and the treatment of neurodegenerative diseases, depression, and acute kidney injury 150. When exposed to an aqueous solution, BP decomposes into phosphate and phosphates, which extract calcium ions from the physiological environment to promote mineralization and facilitate bone tissue formation. Furthermore, the photothermal effect accelerated the degradation of BP and facilitated calcium phosphate deposition. Hence, the combination of BP nanocomposite scaffolds and a mild NIR-based photothermal strategy is expected to produce a synergistic effect that promotes bone regeneration.

A previous study reported the effectiveness of a composite hydrogel against wound infections. The hydrogel, consisting of lauric acid-grafted chitosan, dibenzaldehyde-modified polyethylene glycol, and curcumin-loaded polydopamine nanoparticles, exhibited potent antimicrobial effects. This activity was attributed to the synergistic effect of NIR light triggering, on-demand CUR release, and heat treatment 151. Nonetheless, complete eradication of biofilms requires NIR irradiation at 70°C. Although localized high temperatures can be tolerated by the body for short periods, there is a risk of damage to the surrounding normal tissues 152. Photothermal stimulation at milder temperatures resulted in fewer adverse reactions, but antimicrobial and antibiofilm activities were significantly diminished. Therefore, it is crucial to explore the synergistic effects of photothermal stimulation with different antimicrobial agents and investigate additional combinations of photothermal agents to identify the optimal temperature that maximizes biofilm inhibition and promotes bone regeneration and osseointegration. This issue must be addressed to pave the way for further advancements in the field.

#### 3.2.5 Pressure stimulation

Negative pressure wound therapy (NPWT) is a widely used surgical technique that promotes wound healing. NPWT devices utilize suction to create a partial vacuum, and their effectiveness can be attributed to several primary mechanisms of action 153. These include extracting extracellular inflammatory fluids, blood, and debris through vacuum suction, bringing the wound edges closer together, and facilitating the formation of granulation tissue. This evidence supports the positive outcomes of NPWT in treating open fractures, as it has long been established as an effective treatment for soft tissue injuries. However, the role of negative pressure suction in bone defect repair remains controversial 154. Several studies have demonstrated the potential benefits of negative pressure suction in bone defect repair. The creation of a negative pressure environment around the wound enhances blood circulation and improves the local oxygen supply, thereby facilitating new bone growth and repair. Additionally, negative pressure suction can stimulate local angiogenesis and promote cell proliferation, which aids in the colonization and activation of bone cells. Nevertheless, there are still findings that challenge the effectiveness of negative pressure suction in bone defect repair. Certain studies have indicated that it may have a negative impact on angiogenesis during the healing process, resulting in unfavorable vascular remodeling and new bone growth 155. Moreover, the application of negative pressure suction for bone defect repair poses both technical and operational difficulties and inherent limitations.

Once bone injury occurs, MSCs from various tissues, such as the bone marrow, periosteum, vessel wall, muscle, and circulation, can migrate and differentiate into osteoblast lineage cells to participate in bone regeneration. One study revealed that the osteogenic potential of MSCs can be modulated by mechanical stimulation. Mechanical cues and appropriate stiffness can promote *in vitro* and endochondral osseointegration and differentiation of MSCs 156. Several *in vitro* and *in vivo* studies have demonstrated that bone regeneration can be accelerated by enhancing MSC proliferation, osteoblast differentiation, and osteogenesis-related cytokine expression through continuous or intermittent NPWT. Another study revealed a novel autophagic axis through which negative pressure promotes osteoblast differentiation and bone regeneration in MSCs. Few studies have focused on the fluid flow conditions in the bone marrow cavity and the effect of fluid dynamics on bone formation. The evaluation of fluid flow in the bone marrow cavity induced by negative pressure indirectly confirmed the osteogenic effect of pressure stimulation. However, the mechanics of fluid and trabeculae in the bone marrow cavity under negative pressure have not been thoroughly examined. This challenge arises from the lack of a direct method for detecting fluid flow conditions deep in the marrow cavity, which is encased by cortical bone and soft tissue 157. Therefore, the development of a realistic model of interstitial fluid flow in the bone marrow cavity could aid in understanding the effects of NPWT on trabecular tissue and potentially optimize the protocol of NPWT for bone defects caused by open fractures.

The effect of negative pressure on bone defect repair can be modified by adjusting the working distance and pressure magnitude. As the distance increases, the pressure, shear stress, and fluid velocity of bone trabeculae decrease exponentially, and the magnitude of negative pressure greatly influences fluid dynamics, especially when the pressure is near the pressure source 158. Moreover, the structural anisotropy of bone trabeculae is associated with the anisotropic hydrodynamic behavior of the marrow, and the stimulation of negative pressure leads to unstable marrow flow during trabecular regeneration. When an open fracture occurs, bone tissue is rich in blood supply, but it sustains damage along with the trophoblastic vessels, lymphatic vessels, and soft tissues. The bone trophoblastic vessels and lymphatic vessels are connected to the circulatory system, allowing fluid to enter the bone marrow compartment. Furthermore, the intraosseous pressure (pressure within the marrow chamber) can reach tens of mmHg, making NPWT effective. Pressure gradients are the driving force behind fluid movement, with higher pressure gradients resulting in higher flow rates 159. Studies have demonstrated a significant increase in the pressure gradient in regions near the NP source, which consequently leads to an increase in the fluid flow rate. Consequently, the shear stress of the fluid acting on the trabecular bone is greater when the pressure source is closer. It is important to note that when developing fluid‒solid CFD simulations, the size of the fluid domain should match that of the trabecular specimen (e.g., a 3 × 3 × 3 mm bone specimen surrounded by a 3.4 × 3.4 × 3.4 mm fluid domain). The nature of bone marrow flow induced by negative pressure stimulation is characterized by its permeability 160. Porosity is a commonly used parameter to predict the permeability of trabecular tissue. As the porosity decreases, the BV/TV ratio increases correspondingly. When the BV/TV ratio approaches nearly 100%, fluid flow tends to be obstructed. Consequently, as bone regeneration progresses, fluid flow becomes thinner, and the pressure and shear stresses on the trabeculae diminish. In summary, a negative pressure magnitude of -120 mmHg may have the optimal potential to promote osteogenesis, but the effective working distance may be limited to a specific depth, necessitating further investigation.

Biophysical stimulation has become an integral part of current osteoporosis treatment and is commonly used in clinical practice for conservative interventions such as surgical procedures for bone tissue engineering or physical therapy (**Table** 2). In comparison to other forms of biostimulation, biophysical stimulation possesses the advantages of controllability and convenience. For instance, it enables the regulation of electric current or magnetic field intensity to influence the effectiveness of electromagnetic stimulation, thereby facilitating the promotion of this innovative treatment method. Additionally, the combination of multiple biophysical stimulations has demonstrated significant therapeutic benefits, thus presenting immense research potential. Nonetheless, it is important to note that physical stimulation may not fully halt the progression of osteoporosis, unlike other types of stimulation, which primarily aim to alleviate symptoms and enhance patient compliance. Consequently, adequate attention should be directed toward the exploration of biochemical stimulation.

## 4. Topical biochemical stimulation for osteoporotic osteoregeneration and osseointegration

### 4.1 Bioactive functional groups

**Figure 12:**
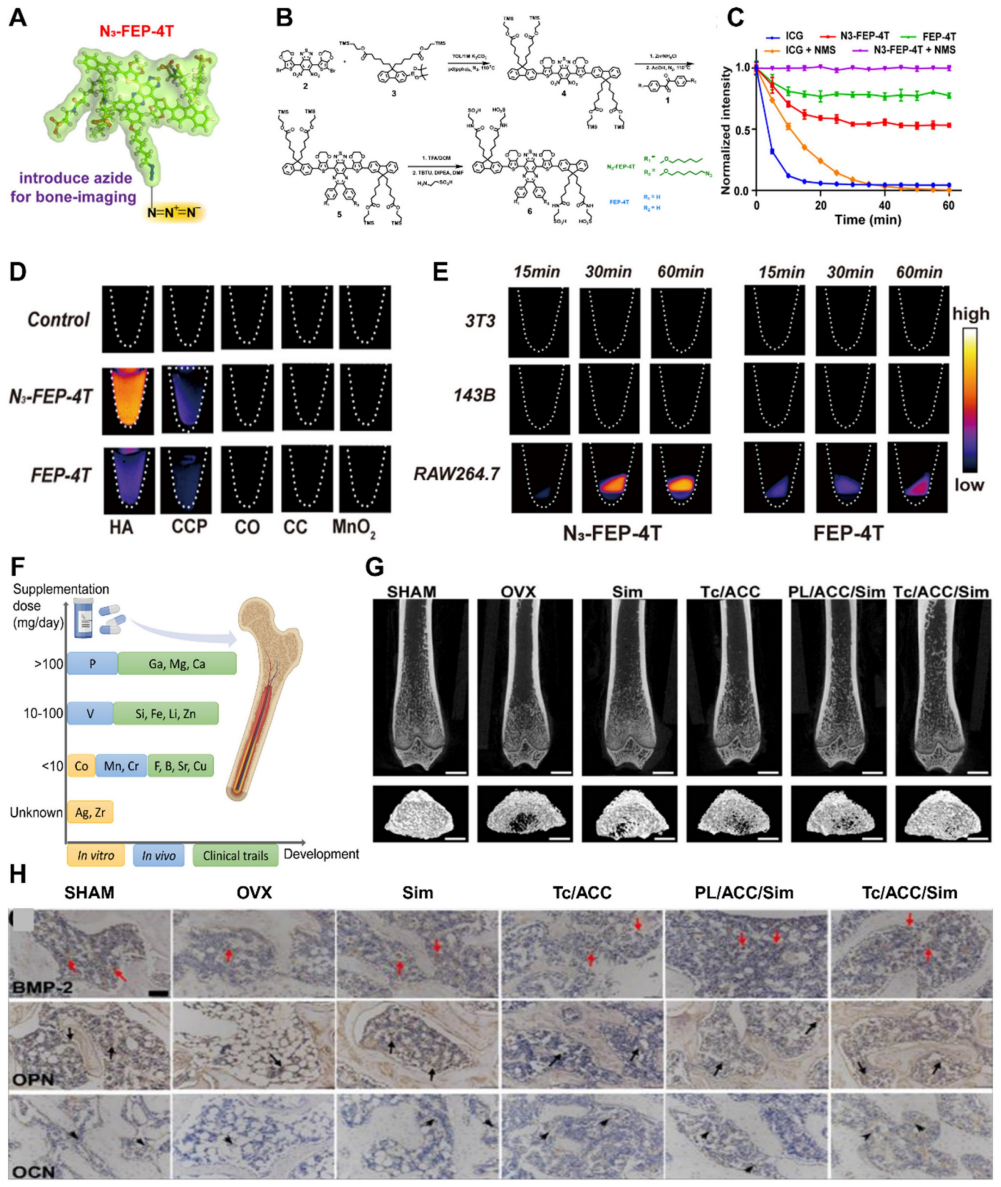
Advances in bioactive functional groups and ion-related research. (A-F) (A) 3D model of N3-FEP-4T. (B) Synthetic route of N_3_-FEP-4T and FEP-4T. (C) Photostability of N_3_-FEP-4T, FEP-4T, ICG in 1× PBS, N_3_-FEP-4T in NMS (normal mouse serum), and ICG in NMS under continuous 808 nm exposure for 1 h at a power density of 0.102 W cm^-2^. (D) Calcium-binding fluorescence image of N_3_-FEP-4T and FEP-4T. (E) Cell binding fluorescence image of N_3_-FEP-4T and FEP-4T. Adapted with permission from 177, copyright 2021. (F) Mineral ion dose ranges. (G) Effect of calcium ions on bone activity and fracture incidence. Adapted with permission from 178, copyright 2023. (H) Immunohistochemical staining of BMP-2 in typical newly formed bone tissue (red arrows) and immunohistochemical staining of the osteogenic markers OPN (arrowheads) and OCN (arrowheads). Scale bar = 100 μm. Adapted with permission from 179, copyright 2020.

A functional group is an atom or group of atoms in a chemical substance that determines its properties. In current clinical treatments, titanium and its alloys are widely used in implantation procedures for osteoporotic bone injuries due to their excellent compatibility, strength, modulus of elasticity, and light weight 161. However, purely metallic materials have limited osteoconductivity, and creating direct bonding at the interface between the material and bone tissue is challenging, often resulting in implant infection, loosening, or the need for secondary surgical removal. Therefore, applying a functionalized coating to a metal surface is a viable option. It is well known that functional groups, as important chemical influences, affect cellular behavior. Compared with surfaces capped with carboxyl (-COOH) and methyl (-CH_3_) groups, surfaces capped with hydroxyl (-OH) and amino (-NH_2_) groups are more conducive to osteogenesis 162. Curran *et al.* demonstrated that biologically active ceramics modified with -CH_3_, -NH_2_, hydroxysulfonyl (-SH), -OH, and -COOH groups induce differential differentiation of MSCs 163. In PEG hydrogels and phosphate-functionalized gels, functional groups promote osteogenesis in MSCs, whereas tert-butyl-functionalized gels promote adipogenesis. The specific mechanism by which functional groups influence cell differentiation remains unclear, and further experiments are necessary to provide detailed evidence (**Figure** 12A-B). Many of the current materials encounter challenges in their integration and uptake, including the hydrolysis of amino borane (NH_3_BH_3_, AB), which necessitates the use of complex metal or nonmetal catalysts. Functional group modulation is an effective approach for enhancing the catalytic activity of AB hydrolysis 164. The proposed mechanisms for catalyzing AB hydrolysis can be categorized into monomolecular and bimolecular types, both involving H_2_O or AB molecules. Theoretically, functional group modulation promotes the catalytic activity of metal NPs and facilitates interactions with H_2_O and/or AB molecules. Notably, the -OH functional group utilizes its high electronegativity and coordination ability to modulate the electronic structure of the metal 165. F plays a crucial role in electrocatalytic oxygen production, as F groups aid in optimizing the activation of the reaction substrate during the reconfiguration process. For example, the exchange between F and hydroxide anions induces the formation of a reactive oxygen (hydroxide) layer, which optimizes the electronic structure and the absorption/desorption energy4@MNF of F-NiMoO, thereby promoting oxygen precipitation reaction (OER) activity 166. Thus, F and -OH groups are considered effective means to enhance AB hydrolysis activity. Several studies have examined the association between the surface functional groups of materials and the adsorption of the protein Fn, as well as the impact of these associations on the behavior of BMSCs. Fn demonstrated substantial adsorption on the surface of the SAM CH_3_ through strong hydrophobic interactions. However, its bioactivity was limited due to the small exposed area and conformational changes in its major cell adhesion sites (RGD and PHSRN). In contrast, the SAMs-NH_2_ and SAMs-COOH surfaces effectively adsorbed proteins through van der Waals interactions, electrostatic interactions, hydrogen bonding, and salt bridges. The adsorption of Fn on the SAM-NH_2_ surface strongly influenced cell adhesion sites, providing favorable conditions for mediating cell adhesion, proliferation, and osteogenic differentiation 167. Although Fn exhibited good bioactivity during adsorption on the SAMs-COOH surface, it showed poor exposure to cell adhesion sites. Nonetheless, it still maintained satisfactory bioactivity under the control of other secondary cell binding sites. In addition, Fn faces challenges in adsorbing onto the surface of hydrophilic SAMs-OH due to the formation of a water film on its surface 168. Although numerous experiments have been conducted to demonstrate the influence of common functional groups, such as hydroxyl, amino, and carboxyl groups, on cell behavior and tissue mineralization, further research is necessary to elucidate the underlying mechanisms and provide detailed comparisons between different functional groups (**Figure** 12C-E). Functional group modulation is a key aspect of treating osteoporotic bone defects, and commonly used functional groups such as -OH and -COOH have widespread clinical applications. The repair of critical-sized bone defects in diabetic patients is a common challenge in osteoporosis treatment. Tao SC *et al.* successfully combined commercial porous β-TCP scaffolds with modularly engineered sEVs that were functionalized using a DSPE-PEG-c (RGDfC) surface and loaded with ZEB1 via EXPLOR technology) immobilized with HA/PLL LbL self-assembled coatings 169. SMSCs generating two recombinant fusion proteins, CRY2-ZEB1 and CIBN-CD9, were connected via flexible connectors (3×GGGGS) for transient docking via blue light manipulation of CIBN and CRY1 to generate sEVs loaded with ZEB2, and *in vivo* and *in vitro* experiments were performed to evaluate their potential to promote bone defect regeneration. The results showed that the enhanced scaffolds exhibited superior immobilization ability for the modularly engineered sEVs, which in turn enhanced angiogenesis and osteogenesis in diabetic injuries and inhibited DM-induced aberrant osteoclast formation 170. This approach has significant potential for the treatment of diabetic and osteoporotic bone defects that are challenging to heal.

BPs, which are stabilized analogs of pyrophosphates, are widely recognized as inhibitors of osteoclastic bone formation. They effectively prevent osteoporotic bone loss and enhance implant osseointegration in patients with osteoporosis. Unlike systemic coatings, BP-doped coatings have the ability to deliver BPs locally, facilitating precise enhancement of osseointegration and bone repair without systemic side effects 171. One team successfully developed an injectable composite colloidal gel for regenerating osteoporotic bone defects. The resulting gel was assembled from bisphosphonate-functionalized gelatin and bioactive glass particles. Following bisphosphonate functionalization, the gelatin nanoparticles demonstrated excellent adhesion to the bioactive glass particles, resulting in elastic composite gels. By carefully adjusting the composition of the composite colloidal gel, they were able to achieve mechanical robustness and self-healing properties. These composite colloidal gels strongly supported cell proliferation and differentiation *in vitro* without requiring additional osteogenic supplements. *In vivo* evaluation confirmed their ability to regenerate osteoporotic bone defects. Additionally, the bisphosphonate modification of gelatin induced therapeutic effects in the surrounding implanted area by enhancing bone density in osteoporotic bone tissue. This offers a new means of functionalizing materials for therapeutic purposes.

Several recent studies have explored the optimal concentration of bisphosphonate groups in coatings. Li *et al.* reported that BPs at a concentration of 10^-6^ M significantly inhibited osteoclastogenesis, with further enhancement observed at 10^-5^ M 172. These studies collectively demonstrated that BPs effectively inhibit osteoclast formation at 10^-5^ M. The results of these studies are summarized as follows. But the inhibitory effect of zoledronic acid was diminished at 10^-4^ M, with no additional dose-dependent improvement observed. A BP concentration of 10^-10^ M promoted the formation of osteoblast-like cells. Low concentrations of BPs have the potential to enhance osteoblast proliferation and osteogenic differentiation, whereas high concentrations significantly inhibit osteoblast activity. In a study by Im *et al.*, BPs at concentrations < 10^-7^ M were found to promote the expression of osteogenic genes and cell proliferation, with the most pronounced osteogenic effects observed at 10^-8^ M 173. Conversely, BPs at concentrations above 10^-8^ M had a significant inhibitory effect on osteoblast activity. Furthermore, BPs inhibited osteoblast proliferation at concentrations above 10^-4^ M. Von Knoch and Lei *et al.* reported that a concentration of 10^-8^ M BP stimulated the proliferation and viability of BMSCs, whereas a concentration of 0.5-1 × 10^-5^ M inhibited BMSC proliferation and osteogenic differentiation 174. Additionally, various concentrations of BPs (ranging from 10^-8^ M to 10^-7^ M) enhanced the mineralization of BMSCs but inhibited the formation of bone nodules at 10^-4^ M. These dual effects can be attributed, in part, to the downregulation of osteogenic genes such as Col, ALP, OCN, and RUNX with increasing BP concentrations. Notably, a reduction in ALP expression can hinder the mineralization process. Therefore, appropriate drug concentrations can effectively block osteoclastogenesis and stimulate the proliferation and differentiation of OBs and stem cells, thereby enhancing the osseointegration potential of implants. Although functionalization offers diverse properties and functions to materials, other factors that should be considered include functionalization side effects, hydrolysis capacity, release kinetics, and pharmacokinetics *in vivo*. The surface functionalization of materials holds great potential for treating osteoporosis, albeit with certain challenges.

### 4.2 Bioactive ions

In addition to employing functional groups for engineered stimuli, the incorporation of various ions on a material's surface can elicit chemical stimuli with beneficial effects. Bioactive ions that promote bone growth and specialized functional ions play significant roles in the processes of bone growth and repair, and their depletion may serve as an indication of systemic bone tissue diseases. However, these ions cannot be directly used as implants for treating osteoporosis 175. Instead, they are commonly combined with matrix materials through coatings, chelation, etc., and subsequently released from these materials during bone damage repair. This release triggers biochemical reactions that can influence tissue metabolism, regeneration, and integration. Additionally, these ions can serve as antioxidants, anti-inflammatories, and antimicrobial agents, indirectly promoting the healing of trauma and tissue regeneration 176. Currently, numerous ions are being utilized or considered for clinical application, and a variety of cells and reactions, including BMSCs, immune cells, and endothelial cells, are involved in the bone repair process. Therefore, it is crucial to explore the interactions and specific functions of ions and different cell types during the early stages of bioactive ion therapy (**Figure** 12F).

The process of bone tissue regeneration and repair includes several key steps, including osteogenesis and mineralization, as well as vasculogenic and neurogenic processes, immune response, and nutrient supply. These factors significantly contribute to the healing of injuries. Several ions play a major role in promoting bone tissue growth, such as calcium (Ca), magnesium (Mg), zinc (Zn), and strontium (Sr), which have been extensively researched and widely used in clinical practice 180. On the other hand, ions such as zirconium (Zr), lithium (Li), and chromium (Cr) are still in the research stage. These ions can promote the regeneration of other tissues; contribute to auxiliary functions such as antimicrobial, antioxidant, and microenvironmental maintenance; and show great potential in the treatment of osteoporotic bone defects. Numerous studies have been conducted to explore specific interventions involving different ions in the bone regeneration pathway 181. Mainstream elements such as Ca, Mg, and Zn are known to promote osteogenesis and inhibit bone resorption through their impact on the Wnt pathway or regulation of the expression of RANK/RANKL. Therefore, these substances are commonly used in clinical applications. Other elements, such as Zr and Co, are still being studied but hold great potential in the process of osteoporotic bone repair. Zirconium, a trace element, is utilized in prosthesis manufacturing due to its exceptional mechanical strength and biocompatibility. Recent studies have shown improvements in bone tissue regeneration with the use of zirconium compounds such as zirconium oxide. These studies highlight that Zr can induce osteoblast proliferation and differentiation, particularly at concentrations ranging from 5-50 μM. Zirconium also activates the BMP signaling pathway and promotes osteoblast differentiation and mineralization 181. However, further research is necessary to fully understand the impact of zirconium on the biological mechanisms associated with bone regeneration and its specific effects on osteoblast behavior. Cu has demonstrated remarkable antimicrobial efficacy in the field of bone regeneration. A scaffolding study revealed that Cu contributes to the activation of β-linked protein signaling pathways and can upregulate osteogenesis-associated gene expression. The study also revealed that Cu is a key component of the bone regeneration process, as it is also a key component of the process. Animal studies suggest that Cu has a downregulatory effect on Wnt signaling, necessitating further research on the effects of Cu^2+^ alone on osteoporotic regeneration. Co^2+^ has been incorporated into various biomaterials, such as CaP coatings, nanoparticles, and bioglass scaffolds. Although not commonly employed in bone regeneration therapies, Co has exhibited a positive effect on Wnt/β-catenin signaling. CoCl_2_, which mimics a hypoxic environment, enhances the expression of β-catenin and Wnt target genes in OBs but inhibits notch signaling 182. Animal testing of codoped bioactive glass scaffolds has shown promising results for use in bone regeneration therapies and for promoting osteogenesis and vascularization.

However, further research is required to determine the safety and efficacy of Co in the treatment of osteoporosis. Fluorine (F) is a highly reactive element with the highest electronegativity and electron affinity among all the elements in the periodic table. Recent studies have demonstrated that when used with hydroxyapatite, F inhibits osteoclast formation and is gradually released from hydroxyapatite. In addition, F has been shown to increase β-linker expression and translocation, which positively affects the Wnt/β-linker pathway and bone. In addition, F has been shown to increase the expression and translocation of β-linker proteins, thus positively affecting the Wnt/β-linker pathway and bone healing 183.

In addition to research on the specific pathways through which ions impact bone cells, precise control of the ion concentration and duration of action can significantly influence osteoporotic bone regeneration. It is important to note that excessive use of these ions may have adverse effects. Extensive research has been conducted to determine the optimal concentration of Ca. The findings revealed that a concentration range of 3 to 10 mM is optimal for enhancing the proliferation of human, porcine, and rat OBs, whereas a concentration between 10 and 20 mM is ideal for stimulating osteogenic differentiation. Combining Ca^2+^ with vitamin D also effectively reduces the duration of fractures in osteoporotic patients 184. Although calcium offers considerable benefits to bone health, it can also present risks, including an increased likelihood of CVD. Excessive concentrations of Ca^2+^ may result in hypercalcemia and an increase in insoluble calprotectin particles. Furthermore, interactions between high levels of Ca^2+^ and platelets can lead to heightened blood clotting. Clinical studies indicate that elevated serum Ca^2+^ levels are associated with carotid plaque thickness, potentially contributing to coronary atherosclerosis and a greater risk of heart disease.

Mg plays a critical role in bone metabolism. By regulating RANK and RANKL, magnesium inhibits osteoclast differentiation, promotes a balance between osteogenesis and osteoblastogenesis, and enhances bone formation 185. Nevertheless, excessive magnesium can be toxic and hinder bone mineralization. It may also decrease tendon reflexes, cause respiratory paralysis, or even result in cardiac arrest. Hence, it is crucial to establish precise, effective, and nonadverse concentrations of Mg^2+^ for bone tissue engineering and endoprosthetic implant production.

In studies focusing on pediatric diseases, Zn deficiency has been shown to negatively affect bone growth, neuronal development, and immune production. Zinc impacts alkaline phosphatase activity during osteoporotic bone regeneration. Research indicates that Zn concentrations between 7 and 20 nM increase alkaline phosphatase activity, which is closely associated with bone mineralization 186. Furthermore, zinc has been demonstrated to inhibit osteoclast formation, making it a promising source of stimulation for osteoporosis treatment (**Figure** 12G-H).

For the application of bone growth-promoting ions and special functional ions that have been proven effective, a common approach is to load these ions within or on the surface of matrix materials and then release them *in vivo* to perform their biological functions 180. Another practice is to use compounds containing related elements as supplements, such as calcium supplements. However, there is a need for further investigation on how this systematic drug can be locally applied and tailored to the individual needs of patients with different degrees of osteoporosis. Additionally, there are numerous bone repair-related ions whose specific mechanisms of action and optimal concentrations for effective results are still unclear. Therefore, additional studies and experiments are required to provide more evidence.

### 4.3 Bioactivity factors

**Figure 13:**
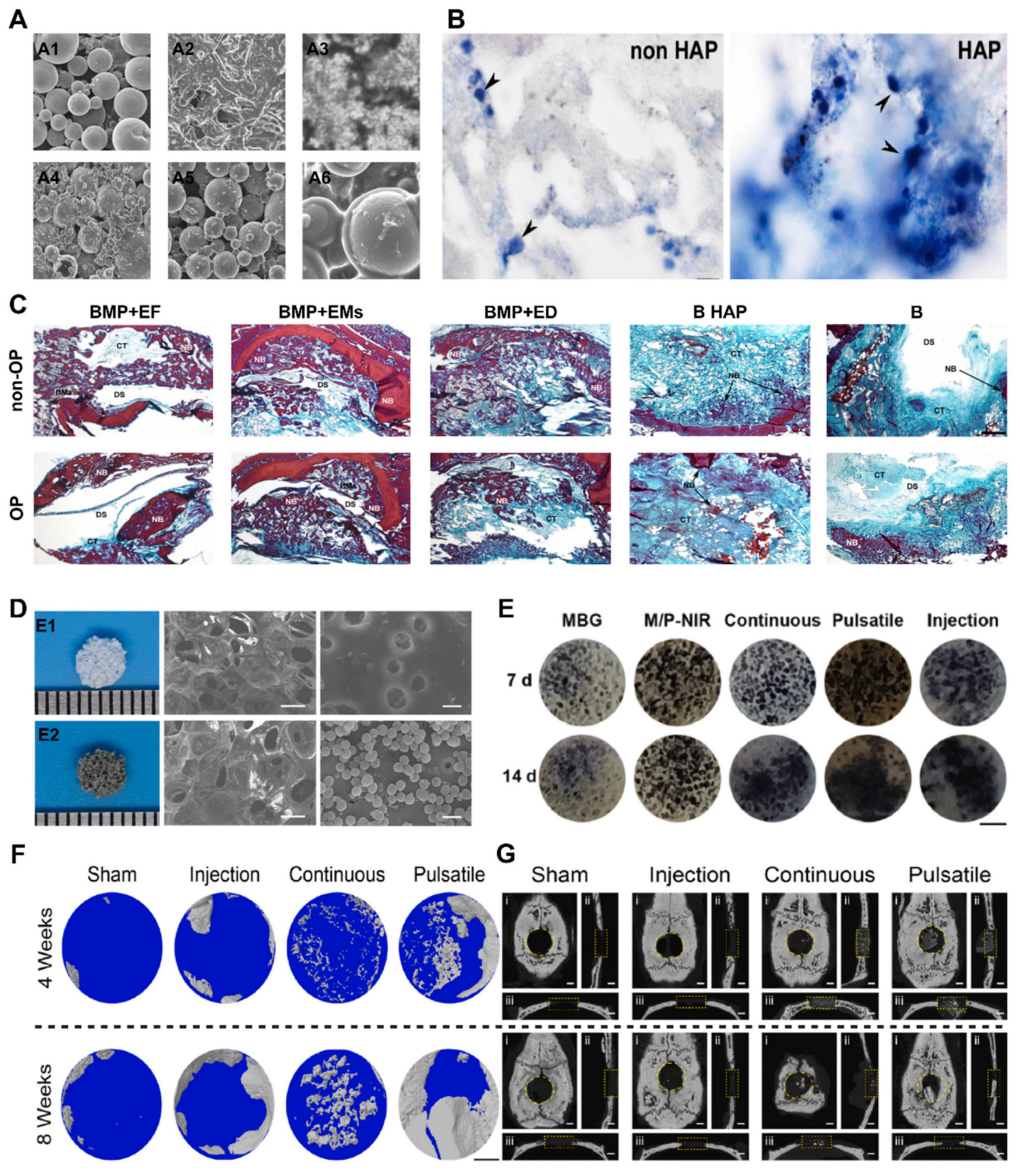
Application and research progress of bioactive macromolecules in osteoporosis treatment. (A) SEM images of microspheres and hydrogels. (B) ALP activity in rMSC cultures. (C) Representative images of cross-sections at the level of critical-size defects in the skull of non-OP and OP rats showing the repair response of different experimental groups at the level of the defects at 12 weeks after implantation. Adapted with permission from 200, copyright 2019. (D) Morphology of the MBG scaffolds and PDA-MBG scaffolds at different scales determined by camera and SEM; scale bars are 200 μm and 1 μm, respectively. (E) ALP activity of BMSCs at different platforms after 7 d and 14 d; scale bars are 1 mm. **p < 0.01, ***p < 0.001. (F) Coronal views of femurs of sham and OVX rats. Micro-CT 3D reconstructed images of defect sites after 4 and 8 weeks of regeneration; scale bar is 1 mm. (G) Micro-CT 2D images of coronary (i), sagittal (ii), and transaxial (iii) slices of the defective region with surrounding tissue after 4 and 8 weeks of regeneration; the scale bar is 1 mm. Adapted with permission from 108, copyright 2023.

In the field of bone tissue engineering, the mainstream choice for mimicking natural bone minerals has been inorganic components such as Hyaluronate (HA) and β-tricalcium phosphate, as they can be incorporated into the materials used 187. However, in many cases, this combination has proven to have difficulty achieving the desired results. To address this issue, organic bioactive factors such as growth factors, antiosteoporosis drugs, or even stem cells can be introduced to these materials 188, 189. Common antimicrobial agents include VEGF, progesterone, BMP-2, bisphosphonates, 17β-estradiol, and MSCs. Various methods exist for loading these biofactors, including direct adsorption onto the material surface. However, the connections formed, such as covalent bonds, between biofactors and materials are often too weak, leading to rapid release of biofactors over a short period. In recent years, there has been a focus on bioabsorbable injectable microspheres, which can effectively fill irregularly shaped defects while minimizing the invasiveness of the surgical procedure, decreasing the amount of pain for patients 190. Biodegradable aliphatic polyesters are commonly used to prepare these microspheres under different conditions, resulting in microspheres with varying surface roughnesses (**Figure** 13A-C).

VEGF plays a crucial role in the bone repair process by enhancing the adhesion and proliferation of endothelial cells *in vitro* and promoting blood vessel formation in the tissue. This highlights the significance of vascularization in osteoporosis treatment, leading to the exploration of combining VEGF with bone implant materials as a highly effective alternative 191. In a study conducted by L. Casarrubios *et al.*, silicon-substituted macroporous HA scaffolds with VEGF-modified surfaces were utilized in a model of corticosteroid-induced limb bone defects in osteoporotic sheep. *In vitro* cell culture tests revealed that nanocrystalline SiHA impeded preosteoblastic proliferation, whereas the presence of VEGF improved endothelial and preosteoblastic biological functions 192. The *in vivo* experiments closely corresponded to the *in vitro* findings, as scaffolds made of crystalline SiHA and modified with VEGF demonstrated remarkable bone regeneration properties, characterized by high ossification, thicker trabeculae, and increased OBs and angiogenesis. Consequently, macroporous scaffolds decorated with VEGF have emerged as suitable bone grafts for bone regeneration, even under severe pathological conditions such as osteoporosis 193. However, a current research challenge involves matching the degradation rate of the material with the release rate of VEGF, necessitating further refinement for optimal concentration maintenance.

BPs are a well-established group of drugs that are used in therapies for metabolic bone diseases. Their main function is to promote bone mineralization and inhibit bone resorption. Studies have shown that BPs induce apoptosis in osteoclasts but prevent apoptosis in OBs, demonstrating their modulatory effect on the osteogenic differentiation of OBs and mesenchymal stem cells 194. Among the family of BPs, alendronate is a nitrogen-containing diphosphate commonly used to treat osteoporosis 195. It induces osteoclast apoptosis by inhibiting farnesyl diphosphate synthase in the mevalonate pathway. In a study, alendronate was doped into microspheres during biomineralization in simulated body fluids (SBFs) 196. Different concentrations (25 μM, 50 μM, or 100 μM) of alendronate were dissolved in 5 volumes of SBF and incorporated into calcium minerals deposited onto the microspheres. This resulted in different release behaviors. Different amounts (25 μM, 50 μM or 100 μM) of alendronate were dissolved in 5 volumes of SBF and doped with calcium minerals deposited onto microspheres, which exhibited different alendronate release behaviors 197. The cell culture results showed that microspheres prepared with 50 μM alendronate had the strongest promotional effect on osteogenic differentiation. However, the adverse effects on cell proliferation and viability were not significant. This finding suggested that the BMP signaling pathway may be one of the mechanisms by which alendronate exerts its activating effect. In *in vivo* experiments, alendronate-loaded microspheres were subcutaneously injected into the dorsum of rabbits for evaluation 198. Histological and immunohistological analyses were performed to assess ectopic neovascularization and osteogenesis 199. The incorporation of alendronate dramatically enhanced the ability of the biomineralized microspheres to promote osteogenesis. In recent years, research has focused on the effect of biofactors in different osteoporosis models and the localized slow release of drugs to achieve on-demand drug release (**Figure** 13E-G).

## 5. Conclusion and outlook

Given the inefficacy and complex adverse effects of current systemic osteoporosis treatments, there is a growing need for localized treatment options. To address this issue, it is crucial to understand the pathological changes in the osteoporosis microenvironment and propose targeted therapeutic targets. By combining these targets with engineered stimulation, a more efficient and personalized therapeutic strategy for the local treatment and remission of osteoporosis can be developed. Bioengineered stimulation consists of two components: biophysical stimulation and biochemical stimulation. Future research on biophysical stimulation should focus on optimizing stimulation parameters and precise and targeted stimulation and exploring its joint application with other therapeutic methods. This research should also aim to translate these findings into clinical application. On the other hand, future research on biochemical stimulation should focus on the development of new growth factors and cytokines, the improvement and development of biomaterials, and their translation into clinical application. Additionally, research should explore the use of inflammation and immunomodulatory mechanisms, multichemical stimulators, and genes involved in biochemical stimulation. Moreover, further exploration of the combined effects of different stimuli is necessary, as the interaction between biophysical and biochemical stimuli can produce synergistic effects that promote more effective osteoporotic bone remodeling and osseointegration.

Although bioengineered stimuli have shown promise in targeting therapeutic targets within the osteoporosis microenvironment, the detailed mechanisms of action and potential adverse effects require further investigation. Additionally, the synergistic effects resulting from the combination of different engineered stimuli need to be explored. Successful localized therapy also hinges on the ability to achieve slow drug release in localized bone tissue, moving beyond systemic therapy. Moreover, investigating whether the therapeutic targets mentioned for treating osteoporosis are common across other systems and the potential for positive interactions between bone and other tissues are key breakthrough points for related technologies. Future studies should aim to refine cellular-level interactions in osteoporosis treatment to the molecular level, explore the synergistic effects of multiple biological factors, and identify optimal parameters for different engineered stimuli to correct pathological changes in osteoporosis.

## Figures and Tables

**Table 1:** Common genomes implicated in the development of osteoporosis

Name	Function Introduction
TGF-β1	TGF-β1 gene polymorphisms are associated with the risk of postmenopausal osteoporosis (PMOP). The TGF-β1/Smads signaling pathway is inhibited in osteoporosis. This signaling pathway is a target for the treatment of osteoporosis.
Bcl-2	Apoptotic factor B-cell lymphoma 2, changes in Bcl-2 gene expression, inhibition of osteoblast proliferation and osteoclast apoptosis.
IL-10	A candidate gene for causing osteoporosis, IL-10 was found to inhibit osteoblast differentiation and interact on osteoclast and osteoblast differentiation in mouse bone marrow cultures.
ACE/CD143	The ACE gene I/D polymorphism may be a genetic factor in osteoporosis.
MMP-13	MMP-13 is one of the most significantly expressed genes in postmenopausal osteoporosis patients. MMP-13 promotes differentiation of osteoclast precursor cells to OBs and indirectly promotes bone resorption
STAT3	Autosomal dominant high IgE recurrent infection syndrome (AD-HIES) is caused by STAT3 mutations and is characterized by eczema, recurrent bacterial infections, and bone and connective tissue abnormalities. Mild trauma fractures and decreased bone density are common symptoms of AD-HIES.

**Table 2:** Biophysical stimulation for osteoporotic osseointegration and osteoregeneration

Source of Stimulation	Category	Function
Endogenous	Stiffness and Elastic Modulus	Materials with higher elastic modulus than surrounding cancellous bone can easily lead to stress shielding. Within this physiological tolerance range, the higher the stiffness and elastic modulus, the more favorable it is for local bone regeneration in osteoporosis.
	Surface Roughness	A higher surface roughness of the material is beneficial for cell adhesion and proliferation. It may have the function of inducing osteogenic differentiation and vascularization.
	Surface Micro/Nanostructure	Surface micro/nanostructure micro/nanofiber structures can simulate the fiber structure in bone tissue, providing better mechanical support and osteogenic biomimetic environment, promoting the attachment, proliferation, and repair of bone defects of bone cells. Micro/nano texture structures can increase surface roughness and fine structures, enhance the mechanical anchoring and interaction between materials and surrounding bone tissue.
	Surface Pore Structure	The three-dimensional pore structure on the surface can provide more surface area and pore structure features, which are conducive to the proliferation and attachment of bone cells, increase the contact area between cells and materials, facilitate the expansion and differentiation of bone cells, guide bone cells to differentiate toward OBs, promote the deposition of bone matrix and bone regeneration, and affect the formation and maintenance of vascularization.
Exogenous	Acoustic Stimulation	Vibration can promote bone cell proliferation and matrix synthesis, increasing bone formation. Sound stimulation utilizes the vibration of sound waves to stimulate bone tissue, which can promote bone cell proliferation, osteogenesis, and angiogenesis. It can be applied around bone scaffolds or implants to promote bone regeneration and integration by transmitting vibration stimuli.
	Electrical Stimulation	Electrical stimulation activates osteogenic signaling pathways by altering cell membrane potential, and electrical stimulation can promote cell proliferation and secretion of bone matrix. Electric stimulation can also promote the production of collagen by bone cells, which is beneficial for the mineralization and hardening of bone matrix. At the same time, electrical stimulation has a certain help in angiogenesis and analgesia.
	Magnetic Stimulation	Magnetic stimulation has similar functions to electrical stimulation. By applying static or alternating magnetic fields, it can alter ion channels and signal transduction within cells, activate signaling pathways within cells, promote cell proliferation, and secretion of bone matrix. Simultaneously, promoting bone matrix deposition and mineralization, as well as angiogenesis and analgesic and anti-inflammatory effects.
	Photothermal Stimulation	Light stimulation utilizes visible or NIR light to stimulate bone tissue, or indirectly affects bone tissue by generating thermal stimulation. It can promote bone cell proliferation and bone matrix production by activating photosensitive pigments or regulating cell metabolism. Moderate photothermal effects can eliminate bacterial membranes, promote blood circulation, increase oxygen and nutrient supply, accelerate bone cell metabolism and synthesis, and promote bone regeneration and integration.
	Pressure Stimulation	The use of negative pressure suction technology can create a negative pressure environment around the wound surface, promote blood circulation and increase local oxygen supply, which helps support the growth and repair of new bones. In addition, negative pressure suction can also increase local angiogenesis and promote cell proliferation, which helps to colonize and activate bone cells.
